# Comparison of Grand Canonical and Conventional Molecular Dynamics Simulation Methods for Protein-Bound Water Networks

**DOI:** 10.1021/acsphyschemau.1c00052

**Published:** 2022-02-11

**Authors:** Vilhelm Ekberg, Marley L. Samways, Majda Misini Ignjatović, Jonathan W. Essex, Ulf Ryde

**Affiliations:** † Department of Theoretical Chemistry, Lund University, Chemical Centre, P.O. Box 124, Lund SE-221 00, Sweden; ‡ School of Chemistry, University of Southampton, Southampton SO17 1BJ, U.K.

**Keywords:** protein solvation, water networks, molecular dynamics simulations, grand-canonical Monte Carlo simulations, grid-based inhomogeneous solvation theory

## Abstract

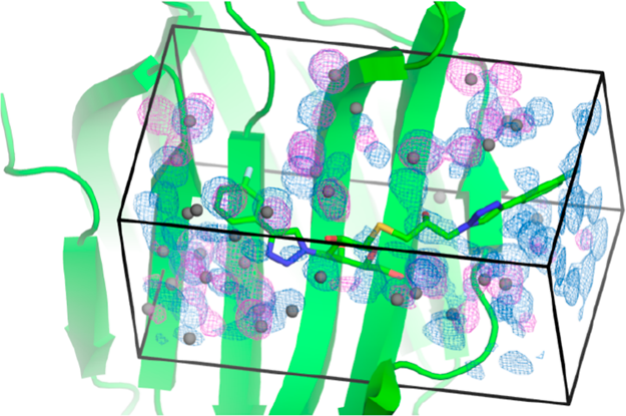

Water molecules play important roles in all biochemical processes. Therefore, it is of key importance to obtain information of the structure, dynamics, and thermodynamics of water molecules around proteins. Numerous computational methods have been suggested with this aim. In this study, we compare the performance of conventional and grand-canonical Monte Carlo (GCMC) molecular dynamics (MD) simulations to sample the water structure, as well GCMC and grid-based inhomogeneous solvation theory (GIST) to describe the energetics of the water network. They are evaluated on two proteins: the buried ligand-binding site of a ferritin dimer and the solvent-exposed binding site of galectin-3. We show that GCMC/MD simulations significantly speed up the sampling and equilibration of water molecules in the buried binding site, thereby making the results more similar for simulations started from different states. Both GCMC/MD and conventional MD reproduce crystal-water molecules reasonably for the buried binding site. GIST analyses are normally based on restrained MD simulations. This improves the precision of the calculated energies, but the restraints also significantly affect both absolute and relative energies. Solvation free energies for individual water molecules calculated with and without restraints show a good correlation, but with large quantitative differences. Finally, we note that the solvation free energies calculated with GIST are ∼5 times larger than those estimated by GCMC owing to differences in the reference state.

## Introduction

Essentially all biochemical processes take place in a water solution. It is well-known that water has unusual properties and has a substantial influence on biochemical reactions, for example, by providing a large dielectric screening, by forming strong hydrogen bond, and by the hydrophobic effect.^1−4^ Consequently, it is important to understand the effect of water molecules in various processes in order to make it possible to manipulate them, for example, in the design of improved catalysts or more effective drugs. Unfortunately, it is hard to obtain such information directly from experiments. For example, calorimetry methods give only the total change in free energies, enthalpies, and entropies, without any possibility to separate contributions from the solvent, whereas crystal structures provide information only about a restricted number of tightly bound water molecules with small dynamics.

Computational methods, on the other hand, can in principle provide atomic-detail information of any process.^1,5,6^ In particular, molecular simulations, obtained with molecular dynamics (MD) or Monte Carlo (MC) methods, can provide a set of ensembles of atomic models for the system of interest, including bulk water molecules. From these, it is rather easy to study the interaction energy (enthalpy) of each water molecule, although the dynamic movement of the molecules may make the interpretation of the results somewhat problematic. However, it is much harder to extract free energies and entropies from the simulations, especially their contributions from individual water molecules. For example, free-energy perturbations can provide accurate estimates of the free energy of a chemical process, for example, the binding of a ligand,^7,8^ but the contributions from individual water molecules or protein residues are more rarely estimated and have a larger uncertainty. Still, the binding free energy of individual water molecules may be calculated, although exchange with bulk water molecules becomes problematic for a solvent-exposed site.^9−11^

Inhomogeneous solvation theory (IST) was developed to obtain local thermodynamic information from molecular simulations.^12−14^ By studying the translation and rotation of the molecules of interest, the entropy can be estimated, which can be subtracted from the average of the interaction energies to give free energies. However, the dynamics and movement of the water molecules are still problematic because a water molecule with a certain role may exchange with bulk water molecules during the simulations. This has often been solved by clustering the water molecules,^15,16^ assuming that molecules that are close in space in the various ensembles have a similar function. However, this is still not fully satisfactory because only water molecules with a high occupancy are considered. Therefore, Gilson and co-workers developed the grid-based IST, GIST,^17^ in which energies and entropies are assigned to small volumes in space, called voxels, instead of specific molecules. Thereby, the interactions and entropies of all water molecules in every snapshot from the simulation are accounted for, giving correct total energies. On the other hand, it is necessary to keep the solute restrained during the simulation to make it possible to properly identify the voxels (relative to the solute) throughout the simulations. With GIST, the average enthalpy, entropy, and free energy for each voxel during the simulation are calculated. Moreover, water densities during the simulations can be calculated, which can be used to identify preferred binding sites of water molecules.

An alternative approach to study water molecules in macromolecular simulations is grand-canonical MC (GCMC) simulations.^18,19^ In this method, in addition to the normal MC movements, attempts are also made to add or remove water molecules from a specific region of the simulated system. With the help of grand-canonical integration,^20^ the optimal number of water molecules in that region can be calculated, as well as the total binding free energy of these molecules (from bulk water), providing binding free energies of complete water networks. Naturally, this approach is most important for hidden binding sites, for which the equilibration with bulk water in standard simulations may be slow. GCMC can also be combined with MD simulations (GCMC/MD) to speed up the exchange with bulk water and therefore improve the equilibration of the simulation, and it can also be combined with alchemical methods to calculate binding free energies^21−25^ Alternative methods with translational MC moves of water molecules from bulk to buried binding sites during MD simulations have also been suggested.^26^

The GIST and GCMC approaches provide partly overlapping results, viz. the free energies of water molecules in a region of interest (ROI) and maps of water densities. Naturally, it is of interest to see how well the predictions of the two approaches agree. Therefore, we here compare the performance of the two methods for two cases: a system with a buried binding site (ferritin) and a system with a solvent-exposed binding site (galectin-3C).

## Theory

### Grand-Canonical Monte Carlo

Protein-bound water molecules often show slow exchange with bulk solvent, which can make such exchanges very slow to equilibrate during computer simulations.^27^ Grand canonical methods attempt to increase the frequency of these events by allowing the number of particles present in a simulation to fluctuate according to a user-specified chemical potential, which is constrained (along with the temperature and volume). This typically involves carrying out MC moves that insert and delete water molecules to/from a predefined ROI, allowing the exchange of waters between this region and bulk water to be accelerated. The acceptance probabilities of these GCMC moves are given by
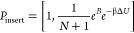

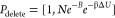
where *N* is the number of water molecules present in the initial state, β = 1/*kT* is the thermodynamic beta (*k* is Boltzmann’s constant and *T* is the temperature), Δ*U* is the potential energy change associated with the proposed move, and *B* is the Adams parameter, which, for convenience, is used as a proxy for chemical potential, and is defined as
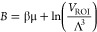
where μ is the chemical potential, *V*_ROI_ is the volume of the ROI, and Λ is the thermodynamic wavelength of water. When the system is at equilibrium with bulk water, the Adams value is given by
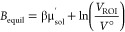
where μ′_sol_ is the excess chemical potential of water and *V*° is the standard-state volume of water—these two parameters depend on the simulation conditions. In this work, these values are taken as −25.9 kJ/mol and 30 Å^3^ for ProtoMS simulations, and as −25.48 kJ/mol and 30.345 Å^3^ for OpenMM—these have been determined in previous work^19,23^ and the differences are likely a result of a number of differences in their calibration, such as software package, configurational sampling, interaction cutoffs, and so forth.

Simulating the system at the equilibrium Adams value will give an equilibrium distribution of hydration sites within the ROI. However, GCMC can also be used to determine the binding free energy of a network of water molecules using titration calculations, where cooperative effects between water molecules are accounted for.^20^ This involves simulating the system at a range of Adams values and recording the average number of waters observed for each value of *B*, from which the difference in binding free energy for water networks of size *N*_*i*_ and *N*_*f*_ is given by
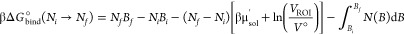
where *B*_*k*_ is the Adams value which produces, *N*_*k*_ water molecules, on average. The sampling of these calculations can be further improved by allowing replica exchanges between simulations at different *B* values.^20^

### Grid-Based Inhomogeneous Solvation Theory

Inhomogeneous fluid solvation theory is a method developed for the thermodynamic analysis of water sites observed in MD simulations.^12−14^ This method calculates the binding free energy of a water site, including the entropic contributions, making use of correlation functions of the translational and rotational behavior of water molecules. This method was reformulated by Nguyen et al. to allow these thermodynamic properties to be resolved onto a 3D grid, rather than water sites, yielding GIST.^17^ This grid-based approach therefore provides thermodynamic properties as a function of the Cartesian coordinates (assuming all simulation ensembles are within the same frame of reference) within a ROI, rather than giving these values only at a finite number of discrete sites.

For this ROI, the total solute–water interaction energy (indicated with the subscript sw) is calculated as the sum over all grid voxels within the ROI
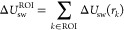
where Δ*U*_sw_(*r*_*k*_) is calculated as the total solute–water interaction energy, averaged over all simulation frames (***r***_*k*_ is the position of voxel *k*). The total water–water interaction energy (indicated with the subscript ww) is somewhat similarly calculated as
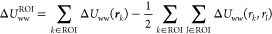
where the Δ*U*_ww_(***r***_*k*_) and Δ*U*_ww_(***r***_*k*_, ***r***_*l*_) terms are calculated as the total water–water interaction energies over the respective voxels, averaged over the number of frames in the simulation.

The total translational contribution to the solvation entropy is determined as the sum of the quantity over all grid voxels within the ROI, as follows
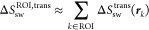

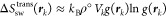

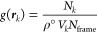
where *N*_*k*_ is the total number of waters observed over all frames within voxel *k*, *V*_*k*_ is the volume of voxel *k*, *N*_frame_ is the number of simulation frames, and ρ° is the number density of bulk water (taken in this work as 0.0329 and 0.0332 Å^–3^ for TIP3P and TIP4P/TIP4PEW water, respectively).^28^ This is based on the approximation that *g*(***r***) is uniform within each voxel.

The orientational contribution to the solvation entropy is calculated as
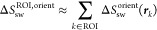

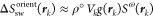

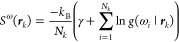
where γ is Euler’s constant and *g*(ω_*i*_|***r***_*k*_) is the value of the orientational distribution for water *i*—this term is calculated as described by Nguyen et al.^17^

Using the terms described above, the solvation free energy of voxel *k* can be calculated as
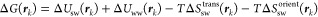
where it should be noted that this value is weighted by the density of the voxel. Further information regarding the underlying theory of GIST can be found in the original publication.^17^

### Simulation Details

**Figure 1:**
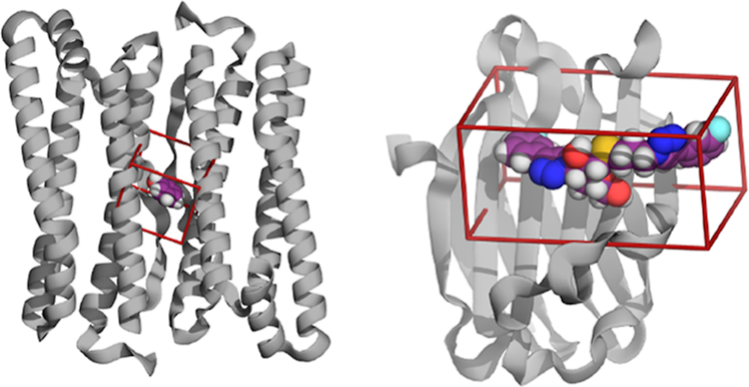
Structures of ferritin (left) and galectin-3C (right) with the respective ROIs (GCMC/GIST boxes) shown. The protein is shown as a cartoon and the ligand in a space-filling model.

Two protein systems were considered in this work—ferritin and galectin-3C. The ferritin system was used to study the timescales required to equilibrate the hydration structure of a buried protein binding site using different simulation conditions and different ensembles. Both protein systems were used to calculate the water binding free energy of a binding site, using both the GCMC and GIST methods, applied to a common ROI, shown for both structures in Figure 1. In all cases, the proteins were represented using the AMBER ff14SB force field,^29^ and GAFF^30^ was used for the ligands, with ligand partial charges determined using the RESP method.^31^ Owing to the large number of simulations performed in this work, the different types of simulations are summarized in this section, with more thorough details provided in the Supporting Information.

### Ferritin

The simulations were based on the crystal structure of a ferritin dimer in complex with phenol bound to the protein (PDB ID: 3F39).^32^ This is called the *holo* structure. In order to study the equilibration times required to fully hydrate the binding site of ferritin, MD simulations were carried out using different software packages (AMBER^28^ and OpenMM;^33^ two packages were used because GIST is implemented in the first and GCMC/MD in the latter) and ensembles (*NVT*, *NPT*, and μ*VT*), with differing simulation conditions, as summarized in Table 1. Additionally, a MC simulation was carried out in ProtoMS to execute a GCMC titration on the binding site. The GCMC box used for the titration was the same as the ROI, with dimensions of 10.9 Å × 14.0 Å × 12.3 Å, centered on the ligand and fixed in space. The GIST analysis of the AMBER MD data used the same box, but with the lengths rounded to the nearest 0.5 Å based on the spacing between grid voxels. GCMC/MD simulations were carried out using version 1.0.0 of the *grand* Python module,^23^ with GCMC sampling of a sphere with a radius of 6 Å, where the center is based on reference protein atoms, chosen to maximize the overlap between this sphere and the cuboidal ROI. All simulations were run for both the phenol-bound structure and for an *apo*-structure, obtained by removing the ligand. There is also a crystal structure of the apo protein (PDB ID: 3F32),^32^ but the backbone root-mean-square deviation (rmsd) between the *holo*- and *apo*-protein crystal structures is only 0.17 Å. The TIP3P water model^34^ was used for all ferritin simulations.

**Table 1:** Brief Summary of the Main Differences between the Four Types of Simulations Carried out for Ferritin

simulation	GCMC	MD	GCMC/MD	MD
software	ProtoMS	OpenMM	OpenMM	AMBER
ensemble	μ*VT*	*NVT*	μ*VT*	*NPT*
simulated system	sphere	cuboidal box	cuboidal box	cuboidal box
nonbonded cutoff/Å	10	12	12	10
temperature/K	300	298	298	298
system charge/*e*	–8	0	0	0
long-range electrostatic treatment	none	PME	PME	PME
spacing between frames	200k moves	12.5 ps	12.5 ps (500 GCMC moves)	10.0 ps
number of repeats	3	3	3	10

### Galectin-3C

**Figure 2:**
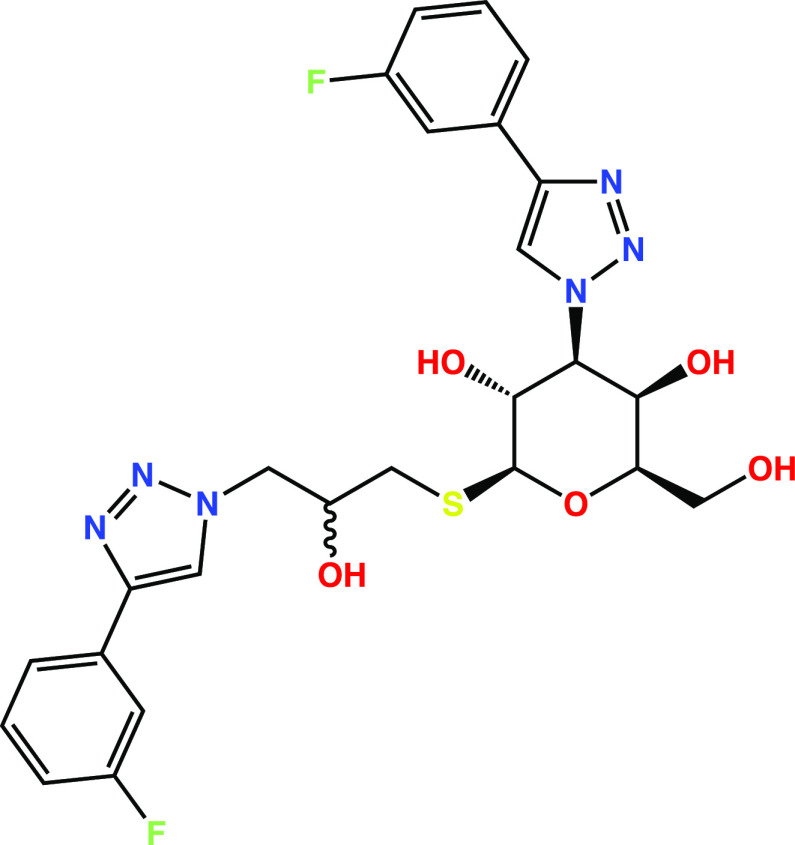
Structure of the ligand simulated in the binding site of galectin-3C. The two diastereomers simulated are referred to as “R” and “S”, according to their stereochemistry at the site drawn ambiguously.

The galectin-3C simulations were run with two diastereomers of the ligand shown in Figure 2, with (R)- and (S)-stereochemistry at the site drawn as ambiguous (PDB IDs: 6QGF and 6QGE, respectively^35^)—these ligands are referred to as R and S in this work according to their stereochemistry at this site. The binding free energy of water to a region around the ligands was studied for this system using both GCMC titration and GIST analysis. Several sets of MD simulations were carried out in order to investigate the impacts of positional restraints on the results obtained using GIST, as detailed in Table 2. The GCMC box was centered around the ligand binding site with dimensions of 26.3 Å × 12.9 Å × 15.0 Å, whereas two different sizes were used for the GIST analysis (to include all conformations of the ligands), as detailed in Table 2, with a spacing of 0.5 Å between voxels.

**Table 2:** Brief Summary of the Differences between the Five Types of Simulations Performed for Galectin-3C^a^

simulation	GCMC	MD (C)	MD (R)	MD (R3)	MD (U)
software	ProtoMS	AMBER	AMBER	AMBER	AMBER
ensemble	μ*VT*	*NVT*	*NPT*	*NPT*	*NPT*
simulated system	sphere	sphere	truncated octahedron	truncated octahedron	truncated octahedron
solvation	TIP4P	TIP4P	TIP4P-Ew	TIP4P-Ew	TIP4P-Ew
restraints on protein & ligand	run with and without constraints	constrained to crystal positions	restrained to crystal positions	restrained to 3–4 ligand conformations	no constraints or restraints
GCMC/GIST box lengths/Å	(26.3, 12.9, 15.0)	(27.0, 13.5, 15.0)	(27.0, 13.5, 15.0)	(30.0, 21.0, 21.0)	(30.0, 21.0, 21.0)
nonbonded cutoff/Å	10	10	8	8	8
temperature/K	300	300 (Berendsen)	300 (Langevin)	300 (Langevin)	300 (Langevin)
system charge/*e*	+4	+4	+4	+4	+4
long-range electrostatic treatment	none	none	PME	PME	PME
spacing between frames	200k moves	1 ps	1 ps	1 ps	1 ps
number of repeats	3	10	10	30–40	10

a Note that the MD–R3 simulations were taken from a previous publication.^35^

## Results and Discussion

### Ferritin Equilibration Times

As described in the Methods section and Table 1, we have performed four sets of MD simulations of a ferritin dimer with or without a phenol ligand. For each simulation, the number of waters, *N*, present within the ROI was measured, and the mean value of *N* at each point in time was calculated as the average over all independent repeats (after aligning each frame to the crystal structure by minimizing the rmsd of the protein C atoms). To model the convergence of hydration, we fit a simple exponential model of the following form
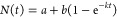
where *N*(*t*) is the mean number of waters present in the ROI at time *t*, and *a*, *b*, and *k* are positive fitted parameters. The model is such that at *t* = 0, the number of waters is *a*, and as *t* goes to infinity, the number of waters will converge toward (*a* + *b*). From these parameters, we then calculate the equilibration time, *t*_eq_, as the value of *t* at which *N*(*t*_eq_) = 0.95(*a* + *b*). From this, the equilibrated mean number of waters in the ROI, *N*_eq_, was calculated, using only the values of *N*(*t*) for which *t* ≥ *t*_eq_.

**Figure 3:**
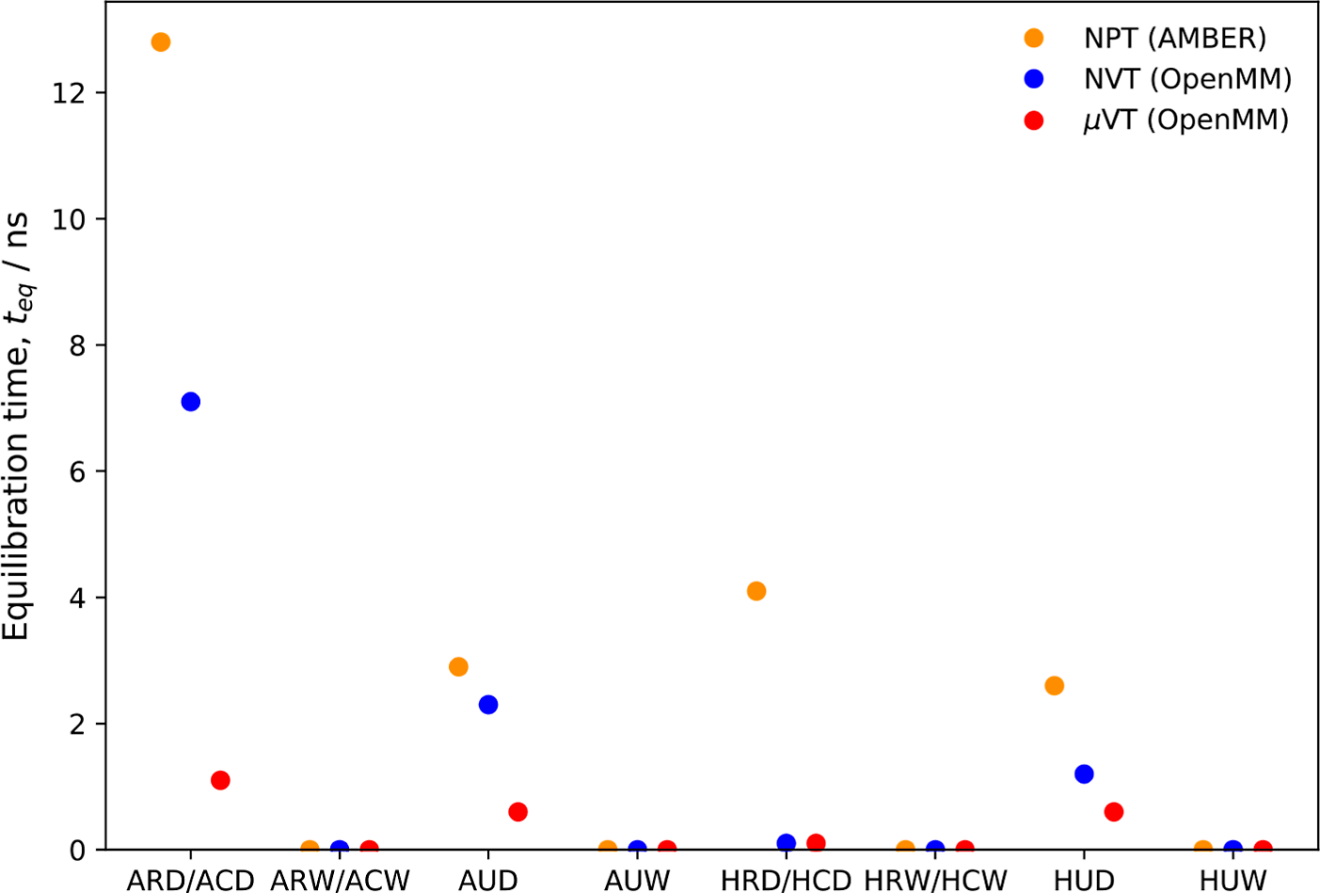
Equilibration times for the various ferritin simulations.

The values determined for these parameters for all MD simulations of ferritin are listed in Table S1, and the equilibration times are presented in Figure 3 (some example fits are shown in Figure S1). The different simulation types are described by three letters: A/H (*apo* or *holo*), C/R/U (constrained, restrained, or neither), and W/D (wet or dry, i.e., with or without water molecules in the ROI at the beginning of the simulation). For example, ARD-*NPT* refers to a restrained *NPT* simulation of the *apo*-structure, starting from a dry binding site.

Several observations can be made regarding the equilibration times presented in Figure 3. First, all dry simulations are slower to equilibrate than the corresponding wet simulations—the latter all appear to be instantaneously equilibrated. This observation is not surprising, given that a certain amount of time is required for waters to diffuse to the ROI from the bulk. Second, the GCMC/MD simulations appear to equilibrate significantly faster than the *NVT* and *NPT* simulations. Interestingly, it also appears that the *NVT* simulations carried out in OpenMM equilibrate faster than the AMBER *NPT* simulations in each case—the reason for this is unclear.

Apart from the ARD-*NPT* and ACD-*NVT* simulations, all systems reported here appear to be equilibrated within 5 ns. The equilibration times for these two simulations (carried out in AMBER and OpenMM, respectively) were 12.8 and 7.1 ns. However, the analogous GCMC/MD simulation (ACD-μVT) equilibrated in just 1.1 ns, which still was the longest equilibration time for any of the GCMC/MD runs.

### Ferritin Water Sampling

**Figure 4:**
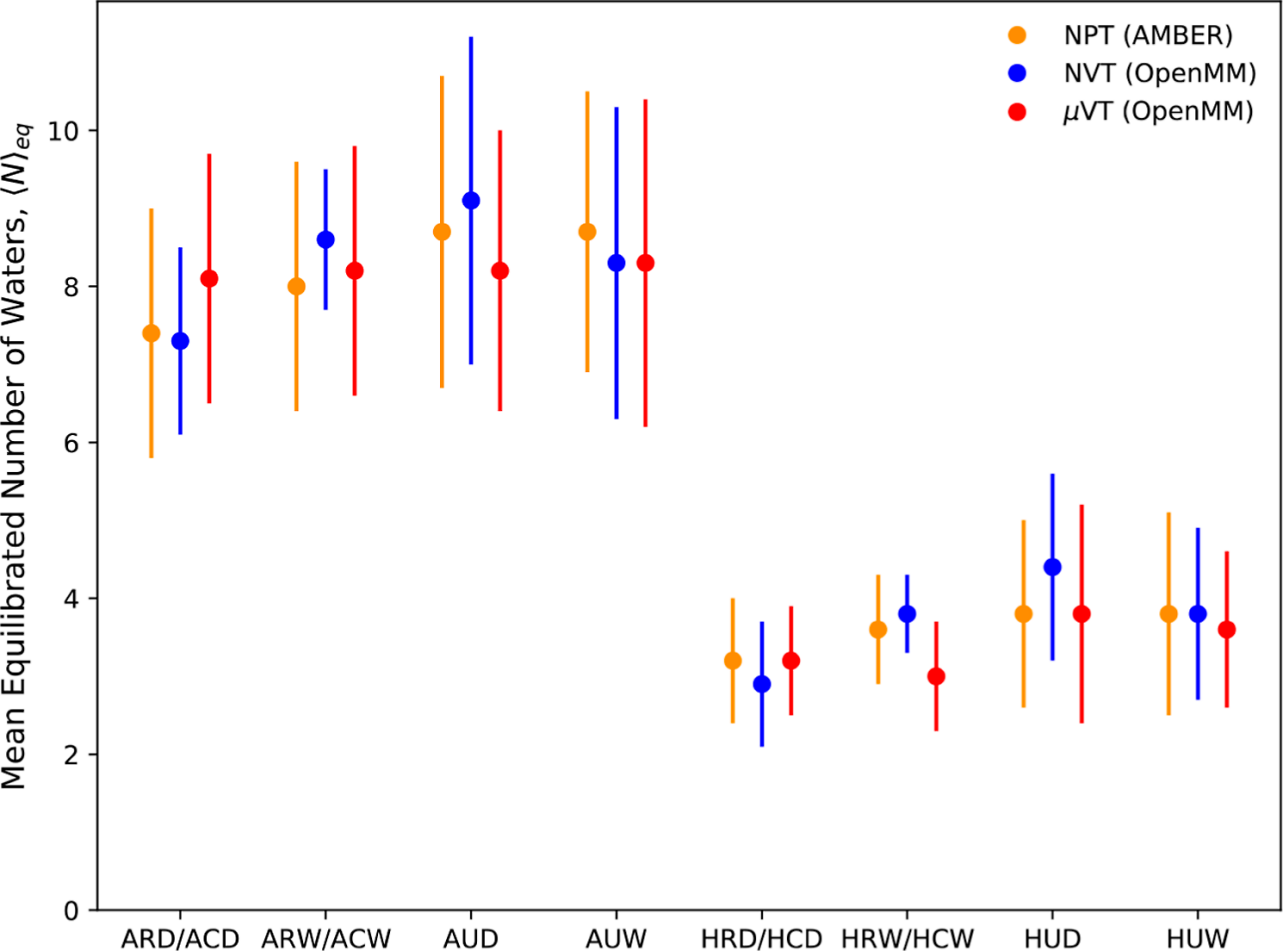
Average number of water molecules observed within the ROI for the various ferritin simulations after equilibration. The error bars represent the standard deviation (in order to demonstrate the distribution sampled) in the distribution of *N*—the number of waters in the region at each point in time, averaged over the independent repeats.

Having found the equilibration times for each of the sets of ferritin simulations, we now look at the differences observed in the water sampling after equilibration. The values of *N*_eq_, for the various ferritin simulations, are shown in Figure 4. It can be seen that the agreement between the different simulation approaches appears to be qualitatively quite good. Moreover, the number of water molecules in the ROI is approximately twice as many in the *apo* simulations than in the *holo* simulations, showing that the phenol ligand displaces approximately four water molecules.

**Figure 5:**
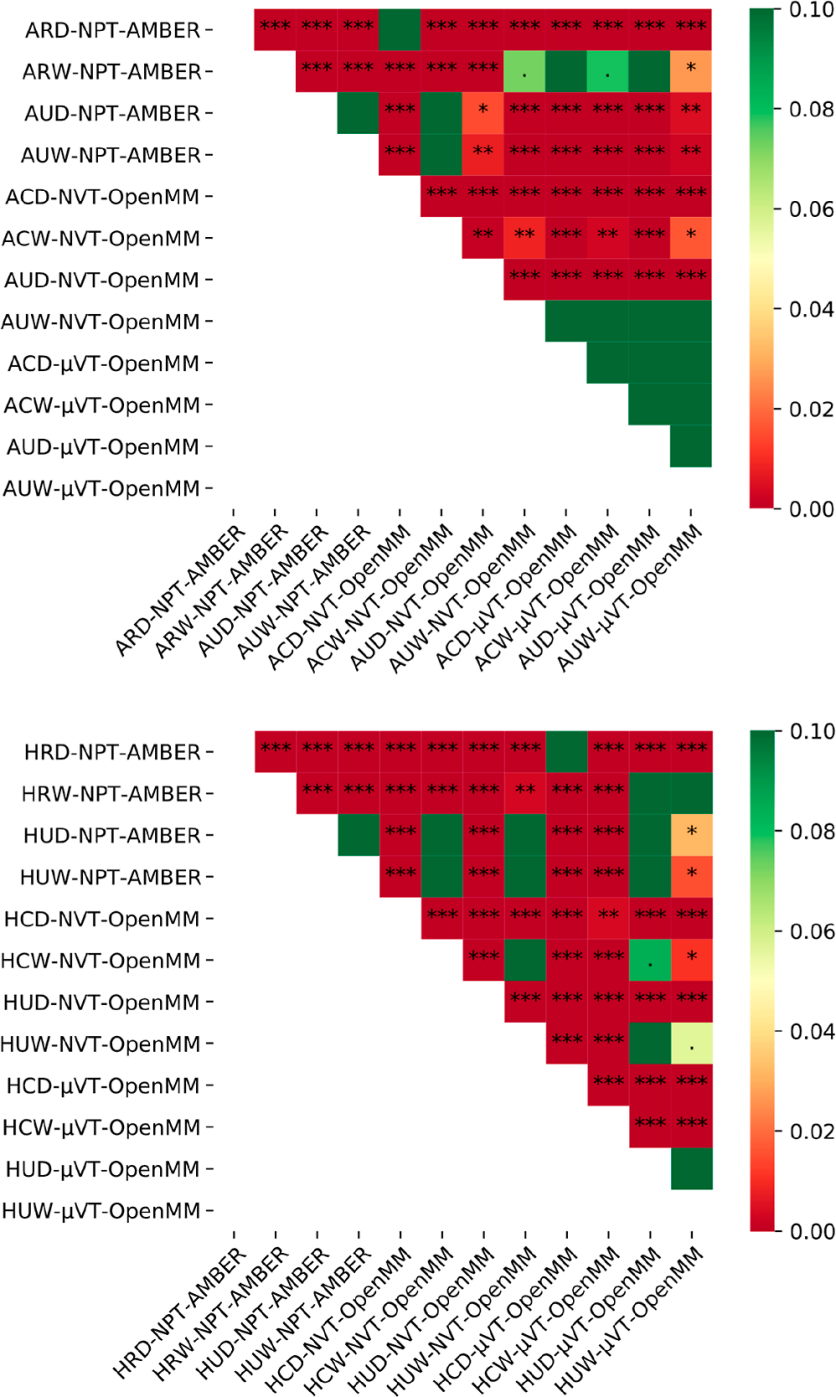
Heat maps showing the *p*-values obtained from the pairwise comparisons of the distributions of the number of water molecules found within the GCMC/GIST region for both the *apo* (top) and *holo* (bottom) data using the Kruskal–Wallis rank test. The null hypothesis is that the two sets of data are drawn from the same distribution, and the significances of the different values are marked as: *p* < 0.001: “***”, *p* < 0.01: “**”, *p* < 0.05: “*”, and *p* < 0.1: “.”. The color scale is capped at 0.1 to highlight differences near the significance threshold of *p* = 0.05.

However, we can carry out a more quantitative assessment, using the distributions of the raw *N* values, post-equilibration (sampled every 250 ps). In order to determine whether or not the sampled distributions of *N* are equivalent, a pairwise analysis was carried out using the Kruskal–Wallis rank test, from which we extract a *p* score for each comparison. This value indicates the probability that the two sets of sampled data are drawn from the same underlying distribution for each comparison, with the result shown in Figure 5.

The initial observation from this statistical analysis is that the GCMC/MD simulations for the *apo*-structure agree very well with each other. For the conventional MD data, good agreements tend to be observed when the data share a common feature regarding either the level of sampling allowed or the initial hydration of the binding sites—for example, unconstrained simulations seem to agree fairly well, as do simulations started with wet binding sites. For the *holo*-data, the majority of the comparisons indicate that the data are significantly different—this may be a feature of the fact that these distributions are much more discrete, given that the value of *N* is primarily distributed between 0 and 5. In any case, these data indicate that for both *apo*- and *holo*-simulations (especially when GCMC sampling is not employed), the sampling of the waters within the ROI is significantly impacted by the system setup and simulation protocol, even after the number of waters in the ROI appears to be equilibrated.

As well as the number of waters observed in the ROI, we can compare the positions of the waters sampled. The water positions for each simulation performed were clustered (based on the oxygen atom) using average-linkage hierarchical clustering, with a distance cutoff of 2.4 Å (the distance between waters present in the same frame was set to an arbitrarily high value to prevent them being clustered together). Using these cluster positions, we can compare the results from two simulations by calculating the Tanimoto similarity of the clusters
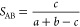
where *a* is the number of clusters in simulation A, *b* is the number of clusters in simulation B, and *c* is the number of clusters which agree between the two sets (within 1.4 Å). We compare all sets of simulations against each other, considering only clusters present for at least 30% of the simulation frames (considering only the equilibrated portion)—this occupancy cutoff was empirically found to give approximately the same number of clusters as the average number of waters observed in the ROI. In each case, all independent repeats from one set of simulations were compared against all repeats of another set, generating a similarity value for each comparison, from which the mean and standard deviation of these similarities were extracted. Additionally, a self-comparison can be carried out, for reference, by comparing the repeats from one set against each other.

**Figure 6:**
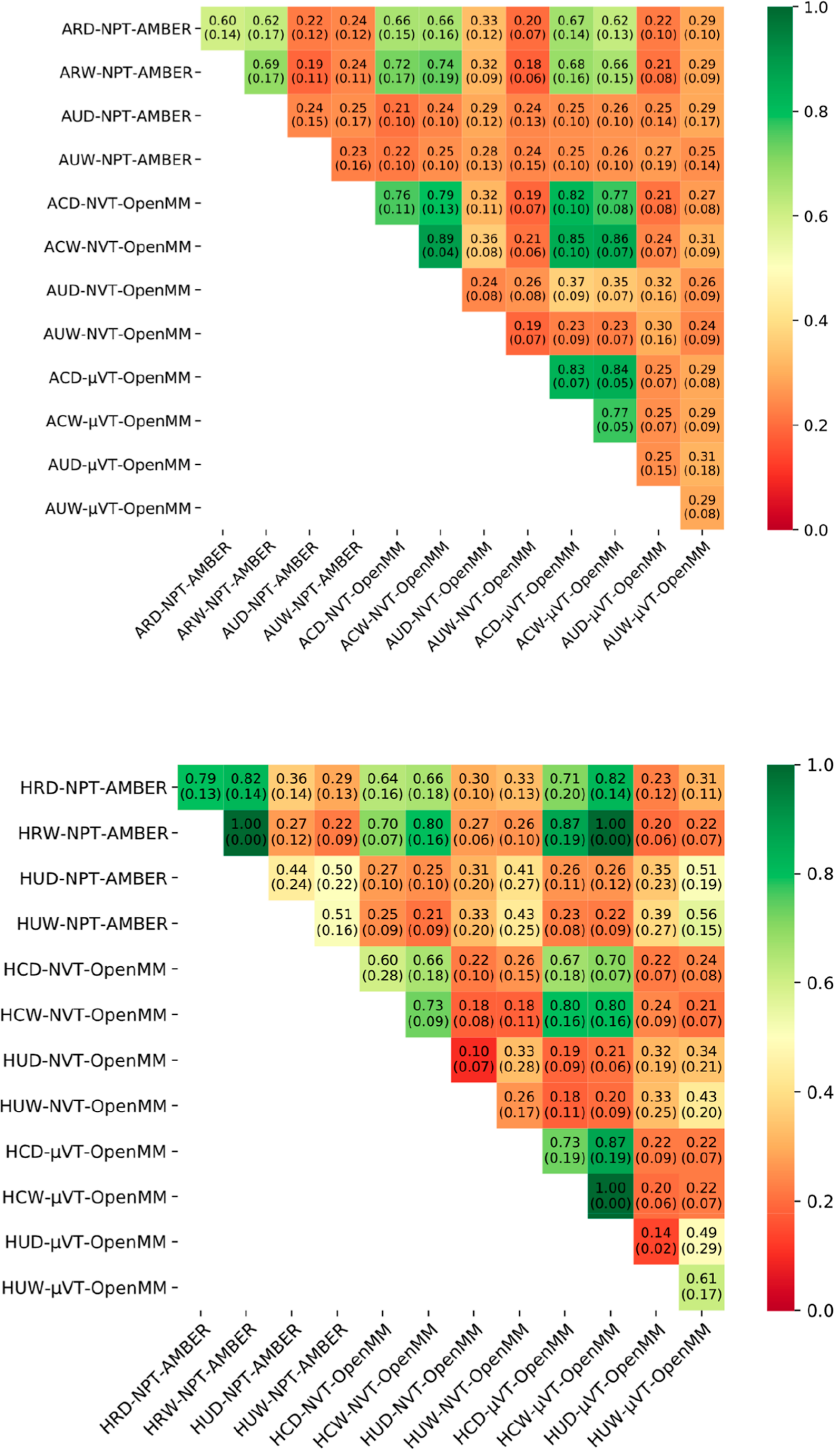
Heat maps showing the Tanimoto similarity results for the clusters determined from the *apo* (top) and *holo* (bottom) simulations. In each box, the mean similarity value from the comparisons carried out is indicated, along with the standard deviation in parentheses.

These results are shown in Figure 6. A clear result here is that the agreement of constrained/restrained simulations with each other is significantly better than comparisons involving simulations with no constraints or restraints. It seems likely that an increase in the motion of the protein and ligand increases the disorder in the water sites, rendering a close comparison of specific water sites more difficult.^36^

### Comparison with Crystallographic Data for Ferritin

The ligand-binding site of ferritin is buried at the interface between two protein subunits. In the crystal structure of phenol-bound ferritin, there are two water molecules close to the ligand, 3.8 Å from the phenol oxygen, 2.7 Å from the sidechain oxygen of Ser27, and 2.3 Å from the sidechain of Arg59 (there are two symmetry-related copies of each residue and also of the water molecule in the binding site).^32^ In the crystal structure of *apo*-ferritin (PDB ID: 3F32), there are four water molecules in the binding cavity (two symmetry-related pairs).^32^ Two of these are close to the positions of the water molecules in the phenol-bound state and they still form hydrogen bonds to Ser27 (2.8 Å) and Arg59 (2.9 Å). The other two water molecules are quite close to the (disordered) positions of the phenol oxygen in the phenol-bound structure. They are 3.2 Å from the other water molecules and do not form any further hydrogen bonds. The symmetry-related pairs of water molecules are 4.6 and 5.1 Å apart.

**Figure 7:**
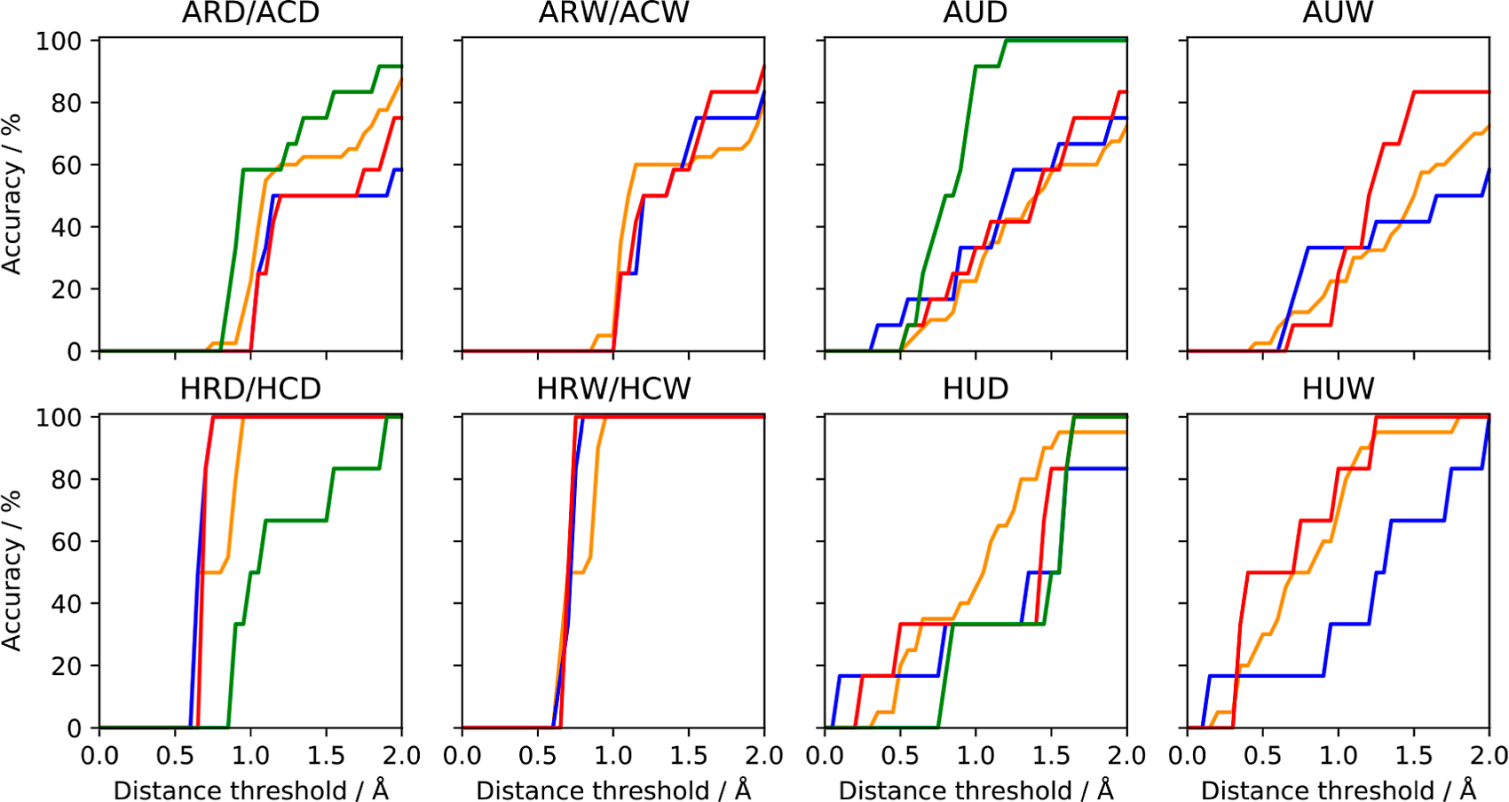
Percentage of the crystallographic waters identified for both *apo*- (top row) and *holo*-ferritin (bottom row) using each simulation type at a range of distance thresholds. For each simulation type, the mean accuracy and associated standard error (over all independent repeats) was assessed by comparing the clusters (with occupancies greater than 30%) with the experimental water sites using a range of distances between 0.0 and 2.0 Å. The color code is as follows: orange: *NPT* (AMBER), blue: *NVT* (OpenMM), red: μ*VT* (OpenMM), and green: μ*VT* (ProtoMS).

Figure 7 shows how well the various ferritin simulations reproduce the positions of the crystallographic water molecules (the percentage of crystallographic waters that are observed within some distance threshold; two snapshots are illustrated in Figure S2). The agreement with the experiment increases with the distance threshold, as can be expected, and the quality of a simulation can be inferred from the area under this curve. Figure 7 shows that the performance of the simulation engines and ensembles varies depending on the type of simulation. For example, the μ*VT* (ProtoMS) simulations gives the best results for the *apo* simulations, but the worst for the *holo* simulations. The μ*VT* (OpenMM) results are mostly among the best for the *holo* simulations. The unrestrained simulations give worse results than the con- or restrained simulations, except for the apo simulation with μ*VT* (ProtoMS). However, it should be noted that this analysis focuses only on the crystallographic waters (two for *holo* and four for *apo*), but all simulations consistently produced around twice as many water sites per frame, as are observed in the crystal structure. An alternative way to compare the simulations with the crystal structures is to study the water densities obtained by the various approaches. This is done for ferritin in Figure S3, showing similar results, but in a less quantitative way.

### Water Structure in Galectin-3C

The ligand-binding site of galectin-3C is a shallow groove on the surface of the protein (cf. Figure 1). The two crystal structures with different ligand diastereomers (Figure 2) are closely similar, with an rmsd for the backbone of 0.13 Å and the two ligands show a similar mode of binding to the protein.^35^ Multiple hydrogen bonds and stacking interactions are observed, primarily involving the galactose half of the ligand with Arg144, His158, Asn160, Arg162, Asn174, Trp181, Glu184, and Arg186. The other half of the ligand forms only two hydrogen bonds to the protein involving the alcohol group of the stereogenic center. Many crystal water molecules are seen around the ligands, some of which form hydrogen bonds to the ligand or the protein (cf. Table S2).

**Figure 8:**
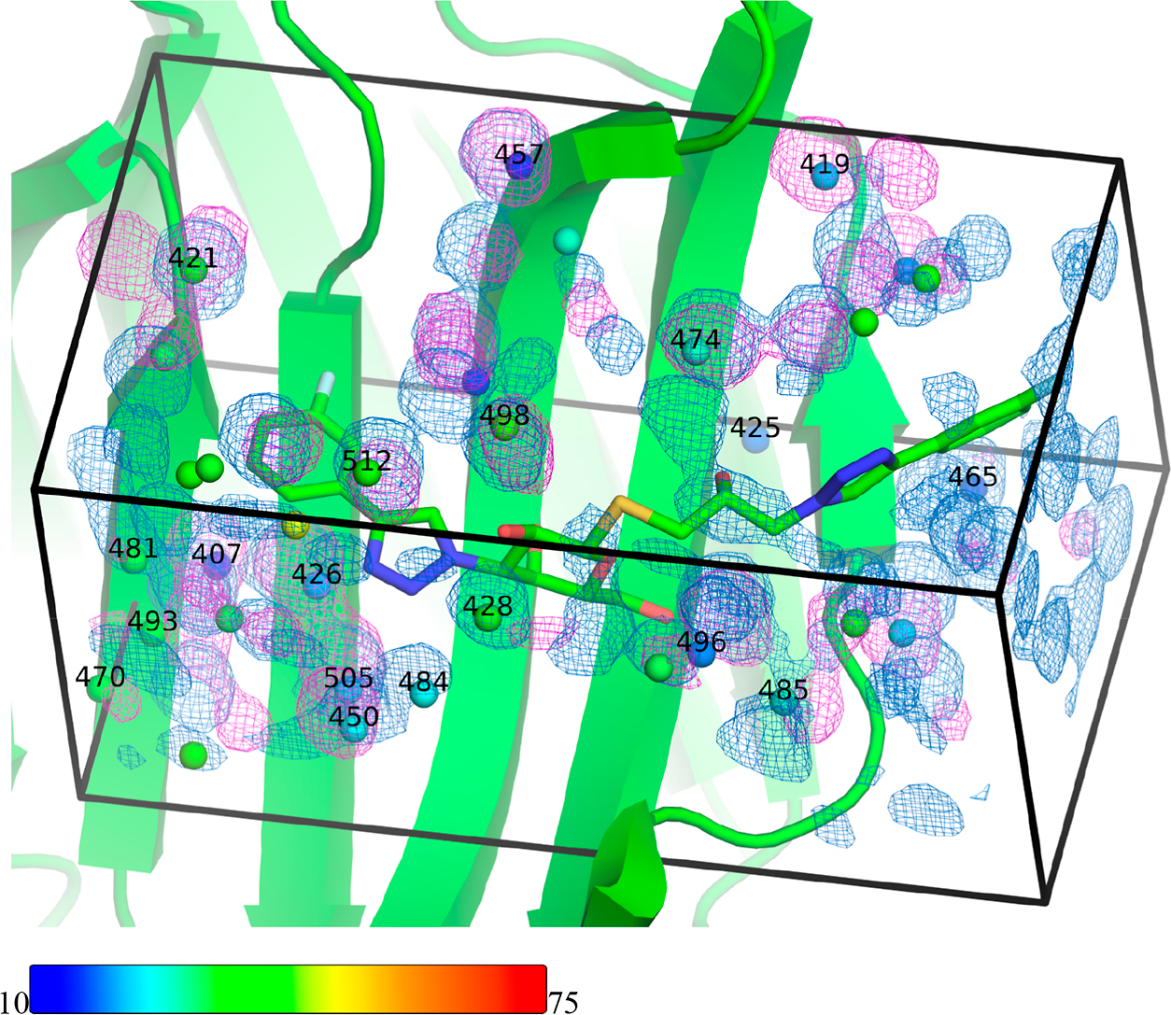
Density maps comparing the water sampling observed using constrained MD (AMBER, marine) or GCMC (ProtoMS, magenta) for the R ligand in complex with galectin-3C. The corresponding figure for ligand S is shown in Figure S4. The protein and ligand are shown with the crystallographic coordinates, and the experimental water sites are colored according to their temperature factors (scale shown at the bottom of the figure). Water molecules that make hydrogen bonds with the protein or the ligand (Table S2) are marked with residue numbers. The density maps are contoured at an isovalue of 0.6.

Because the binding site of galectin-3C is open to the solvent, there is no need to study the equilibration times of the water molecules. Instead, we concentrate on the water structure of the binding site. The water densities from the GCMC and the constrained AMBER MD simulations are compared in Figures 8 and S4 for ligands R and S, respectively. Water molecules that make hydrogen bonds with the protein or the ligand (Table S2) are marked with residue numbers. It can be seen that the localized water molecules found by the two methods agree in general fairly well for both ligands. However, the agreement is far from perfect and many of the densities are significantly translocated between the two calculations. In several cases, both GCMC and MD reproduce the positions of crystal-water molecules well, for example, water molecules 407 and 457 for ligand R (which have low temperature factors). In other cases, only MD (e.g., water 428) or only GCMC (e.g., water 419) reproduce the crystal-water molecules. There are also several crystal-water molecules that are poorly reproduced by both MD and GCMC. This is mostly water molecules without any direct interactions with the protein or the ligand (i.e., without any number in the figure) and with a rather high temperature factor (green, yellow, or red). However, this applies also to water molecules 425 and 470 (for ligand R), which do interact with the protein. For the R ligand, the MD densities reproduce the well-defined water molecules slightly better than the GCMC densities, whereas for the S ligand, the two methods give comparable results.

**Figure 9:**
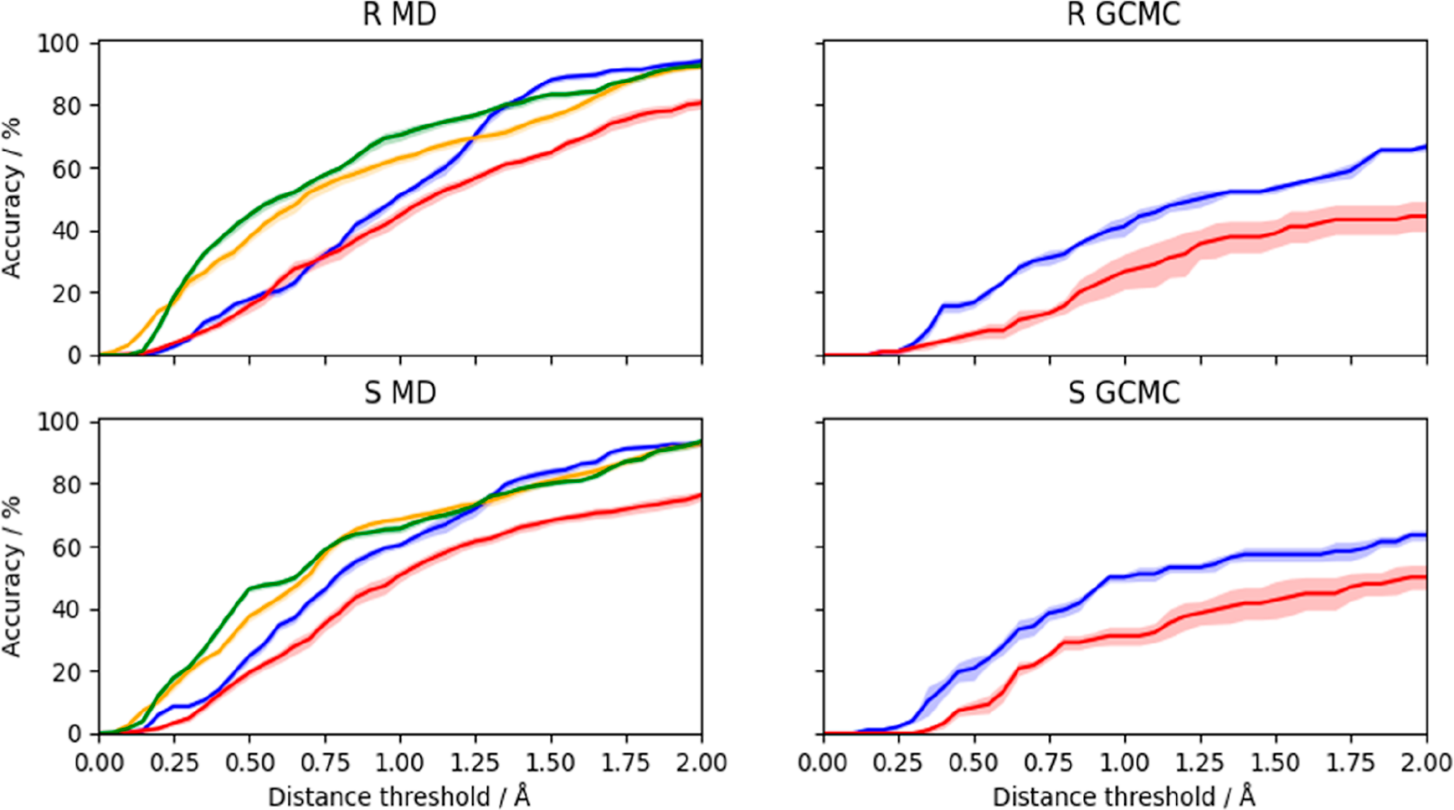
Percentage of the crystallographic waters identified for both R (top row) and S bound to galectin-3C (bottom row) for each simulation type at a range of distance thresholds. For each simulation type, the mean accuracy and associated standard error (over all independent repeats) was assessed by comparing the clusters (with occupancies greater than 30%) with the crystallographic water sites using a range of distances between 0.0 and 2.0 Å. The color code is as follows: blue: C, red: U, orange: R3, and green: R.

A more quantitative analysis is shown in Figure 9, showing how many of the crystallographic waters are reproduced in the MD simulations (cluster centroids with an occupancy larger than 0.3) within a certain distance. It can be seen that the restrained simulations (R and R3) give the best results, reproducing 73–81% of the crystal water molecules within 1.4 Å. The unrestrained simulations (U) give significantly worse results at all distances, which probably reflects mainly the problem of superposing structures with a fully flexible protein.^36^ The constrained simulations (C) give results that are similar to the unrestrained simulations for short distance thresholds, but as good as the restrained simulations at for large thresholds (e.g., 92–94% at 2.0 Å). If instead all water centroids with an occupancy larger than 0.01 are considered, ≥95% of the crystal waters are reproduced within 1.1–1.4 Å for all simulations. The GCMC simulations show a similar trend, but fewer crystal water molecules are reproduced, especially at larger distances (44–50% for U and 64–67% for C within 2.0 Å).

### GCMC Free Energy Analysis

From the GCMC titrations performed using ProtoMS, we can rigorously calculate the binding free energies of the water networks within the ROI. These results are presented for ferritin in Table 3, and the corresponding titration plots are shown in Figures S5 and S6. Reassuringly, the number of waters observed at the equilibrium *B* is in agreement (to the nearest integer) with the number of waters predicted at the free energy minimum. As for the MD simulations, we observe slightly more waters binding to the unconstrained proteins than the constrained simulations. Interestingly, the number of waters predicted in each case is slightly larger than is observed for the corresponding MD simulations (Figure 4 and Table S1). A more significant difference is noted for the unconstrained simulation of the *holo*-structure, where six waters are predicted at equilibrium. This occurs due to a separation of two repelling Arg residues, which creates space for two additional waters. Given that this behavior was not observed in any of the MD simulations, it is probably an artifact of either MC protein sampling or the lack of long-range electrostatic corrections in the MC simulations.

**Table 3:** Results Obtained from the GCMC Titration Analysis of the Both *apo*- and *holo*-Ferritin, with and without Constraints on the Protein and Ligand^a^

structure	sampling	Δ*G*_bind_^°^	optimal *N*	*N*(*B*_equil_)
*apo*	constrained	–90.0 ± 0.8	9	8.7 ± 0.1
*apo*	unconstrained	–109.2 ± 0.8	10	10.2 ± 0.2
*holo*	constrained	–63.2 ± 0.4	4	3.8 ± 0.1
*holo*	unconstrained	–77.4 ± 0.8	6	6.0 ± 0.2

a The table shows the value of the binding free energy minimum (Δ*G*_*bind*_^°^ in kJ/mol), along with the corresponding optimal value of *N*. For comparison, the mean number of waters observed at *B*_*equil*_ is also given (with standard error).

The binding free energies of the binding-site water networks quite closely follow the number of water molecules in the binding site, decreasing from −63 or −77 kJ/mol in *holo*-ferritin to −90 or −109 kJ/mol in the *apo* protein, and also becoming more negative by 14–19 kJ/mol when going from the constrained to the unconstrained simulations.

The analogous results for the titrations of the galectin-3C systems are shown in Table 4, with the corresponding titration plots shown in Figures S7 and S8. For this protein, the number of waters corresponding to the free energy minimum is always one less than the mean number of waters observed at *B*_equil_. This is because the free energy minimum is very shallow (owing to the solvent-exposed nature of this binding site, many of these waters are bulk-like and will have binding free energies close to zero), so the exact minimum is difficult to reliably identify. Again, the unconstrained simulations produce more waters within the GCMC box owing to the increased flexibility of the environment.

**Table 4:** Results Obtained from the GCMC Titration Analysis of Galectin-3C with Both the R and S Ligands, with and without Constraints on the Protein and Ligand^a^

ligand	sampling	Δ*G*_bind_^°^	optimal *N*	*N*(*B*_equil_)
R	constrained	–637 ± 3	81	81.8 ± 0.1
R	unconstrained	–619 ± 3	84	84.8 ± 0.5
S	constrained	–667 ± 2	80	80.7 ± 0.6
S	unconstrained	–594 ± 3	83	84.0 ± 0.6

a The table shows the value of the binding free energy minimum (Δ*G*_bind_^°^ in kJ/mol), along with the corresponding optimal value of *N*. For comparison, the mean number of waters observed at *B*_equil_ is given (with standard error).

As expected, the binding free energies of the water networks (Δ*G*_bind_^°^) are appreciably more negative for galectin-3C than for ferritin, reflecting the much larger number of water molecules in the ROI. However, the binding free energy no longer correlates with the detailed number of water molecules. Instead, it is 18–73 kJ/mol less negative for the unrestrained simulations than for the constrained simulations, although the number of water molecules increase by three for both ligands. It is also interesting to note that when the system is constrained, the water network of the S ligand is 30 kJ/mol more favorable than that of the R ligand, but when the constraints are removed, this swings to −25 kJ/mol. It is possible that the ligand and protein have moved in the unrestrained simulations to optimize their interactions and in doing so the water network is destabilized, while the total free energy of all simulated particles decreases.

### GIST Free Energy Analysis

The total solvation free energies obtained from the GIST analyses of the ferritin MD simulations are given in Table 5, with the individual terms given in Table S3. It can be seen that the total solvation free energies are approximately twice as large for the *apo*-simulations than for the *holo*-simulations. This reflects that the *apo*-simulations have approximately twice as many water molecules in the GIST box as the corresponding *holo*-simulations (Figure 4).

**Table 5:** Free Energy Results (kJ/mol) from the GIST Analysis of the AMBER Simulations of Ferritin^a^

system	Δ*G*^*ROI*^
ARD	–304.5 ± 10.7
ARW	–360.9 ± 11.4
AUD	–459.1 ± 17.0
AUW	–432.7 ± 10.0
HRD	–166.4 ± 3.1
HRW	–181.5 ± 1.9
HUD	–235.7 ± 9.1
HUW	–236.3 ± 9.5

a Δ*G*^ROI^ is calculated as the sum of Δ*G*(***r***_*k*_) for all grid voxels in the ROI.

The GIST analysis is normally performed with the protein and the ligand restrained in order to simplify the alignment of the snapshots and to reduce the uncertainty of the calculated enthalpies and entropies. However, it is possible that the restraints may affect the calculated properties.^4^ The present simulations give us a good opportunity to evaluate the effect of restraints for both absolute and relative GIST properties. The results in Table 5 show that the restrained simulations always give a more favorable total solvation free energy than the unrestrained simulations (by 55–155 kJ/mol). This was also observed for the GCMC data (Table 3) and is likely a result of protein motion allowing the system to reach a more favorable arrangement and allowing for the binding of a slightly larger number of water molecules. Additionally, the uncertainties in the results are slightly larger for the unrestrained simulations, as expected.

Of course, it is problematic if the restrained and unrestrained simulations do not give similar results, as it becomes unclear whether the results of the restrained simulations have any relevance for the true unrestrained system. However, the prime use of GIST is to evaluate the thermodynamic signature of individual water molecules or regions in the binding site, for example, to determine what water molecules are favorable to displace upon ligand binding.^17^ Therefore, we evaluated the enthalpies and entropies of the most occupied water positions in the restrained and unrestrained simulations. The results in Table 6 shows that the correlation of the normalized solvation free energies for the various water sites are quite good (*R* = 0.6–0.9), higher for the *apo* simulations (0.8–0.9) than for the *holo* simulations (0.6; cf. Figure S9). However, for the individual enthalpy and entropy terms (Table S4), the correlation is worse, even negative for Δ*E*_ww_ for the *holo* simulations. Moreover, the mean absolute deviations (MADs) for the individual water sites are 5–11 kJ/mol, with maximum errors of up to 35 kJ/mol. Thus, the results from the restrained and unrestrained simulations agree reasonably in relative terms, but far from quantitatively.

**Table 6:** Comparison between Individual Water Sites in the Restrained and Unrestrained Simulations of Ferritin and Galectin-3C^a^

	*R*	MAD	Max	MRD	*N*_w_
ARD–AUD	0.89	5.4	17.6	0.08	29
ARW–AUW	0.84	8.0	23.1	0.12	39
HRD–HUD	0.59	11.4	35.1	0.12	9
HRW–HUW	0.64	10.7	34.5	0.13	7
RR–RC	0.85	6.4	29.3	0.10	169
SR–SC	0.91	6.9	28.4	0.11	166
RU–RR3	0.88	3.3	40.9	0.05	636
SU–SR3	0.79	3.6	98.5	0.06	628

a The water sites were first identified by clustering of all simulations and then paired between the two sets of simulations based on the distance (only sites with a distance < 1 Å were compared). Then, the normalized GIST solvation free energies for the voxel involving the cluster centroids were compared with respect to the Pearson correlation coefficient (*R*), the MAD, (kJ/mol), the maximum deviation (Max, kJ/mol) and the mean relative deviation (MRD, i.e., the absolute difference divided by the maximum of the two individual absolute values). *N*_w_ is the number of water sites considered in the comparison. Similar results were obtained if we instead considered all voxels within 1.4 Å of the cluster centroids, the voxels with the maximum population within 1.4 Å of the cluster centroids or the voxels with the maximum populations in the GIST box, not closer than 1.4 Å. With randomized water sites, *R* drops below 0.1, whereas MAD, Max, and MRD increase by a factor of 2–3.

The GIST analyses for the various MD simulations of galectin-3C are presented in Tables 7 and S5. As described in the Methods section, we performed four sets of simulations, viz. with the protein and the ligand constrained (C), with restraints to the crystal structure (R) or to 3–4 structures obtained from a clustering of the unrestrained simulations (R3),^35^ or without any constraints or restraints (U). C was designed to be as similar as possible to the GCMC simulations, whereas the others used a MD setup similar to that used for ferritin. Owing to the larger movement of the ligands in the U and R3 simulations, we needed to use a larger GIST box for these simulations (to ensure that the ligand is within the box in all snapshots). Consequently, the total solvation free energies are much larger for those two simulations.

**Table 7:** Free Energy Results (kJ/mol) from the GIST Analysis of the AMBER Simulations Carried out for Galectin-3C^a^

ligand	simulation	Δ*G*^ROI^	*N*
R	C	–3 709 ± 7	87.9 ± 0.1
	R	–4 535 ± 1	80.1 ± 0.1
	R3	–15 010 ± 2	284.8 ± 0.2
	U	–15 281 ± 16	284.9 ± 0.7
S	C	–3 723 ± 10	87.6 ± 0.2
	R	–4 464 ± 2	79.1 ± 0.1
	R3	–14 967 ± 3	284.9 ± 0.1
	U	–15 262 ± 26	283.4 ± 0.8

a Δ*G*^ROI^ is calculated as the sum of Δ*G*(***r***_*k*_) for all grid voxels. The average number of water molecules in the ROI is also given for each simulation. Note that two different sizes are used for the ROI: one for the C and R simulations and another for the U and R3 simulations.

From the results in Table 7, it can be seen that the U simulations give appreciably more negative solvation free energies than the R3 simulations. Likewise, the R simulations give more negative solvation free energies than the C simulations, following the trends observed for ferritin. However, in the latter case, the average number of water molecules in the GIST box is actually lower in the R simulations than in the C simulations, showing that the more negative free energy is not a trivial effect of the number of water molecules.

It could be expected that the difference in the solvation free energies between the R and S ligands would be more consistent in the various simulations. However, we still observe large differences between the various simulations: Δ*G*^ROI^(*R*) – Δ*G*^ROI^(*S*) = 14 ± 12 kJ/mol for C, but −71 ± 3 kJ/mol for R and −42 ± 3 kJ/mol for R3. The U simulation gives such a large uncertainty (−19 ± 30 kJ/mol) that it might agree with any of the other simulations.

If we consider the individual water sites (Table 6), we obtain results similar to those of ferritin: The correlation for the total solvation free energy is high (0.79–0.91; cf. Figure S9) and the mean relative errors are small (5–11%). However, the MAD and maximum errors are large, 3–7 and 28–98 kJ/mol. Still, the correlation coefficients are quite large for all entropy and enthalpy components, 0.52–0.96 (Table S3). The results are somewhat better when comparing the R and C simulations than the U and R3 simulations because the R simulation is restrained, not fully unrestrained.

A comparison of the results in Tables 3–5 and 7 shows that the water free energies calculated with GCMC and GIST do not agree—the GIST free energies are 3–6 times larger in absolute terms. Both free energies roughly increase with the number of water molecules in the ROI, but the free energy per water molecule is also 3–8 times larger for GIST (−41 to −62 kJ/mol) than that for GCMC −7 to −17 kJ/mol). The reason for this is that they measure different free energies. The GCMC titrations calculate the standard-state binding free energy of a network of *N* waters within the ROI, where the reference state is the ROI with no waters present. On the other hand, GIST estimates the solvation free energy of each voxel, where in the reference state, that voxel is dehydrated but all others are hydrated (i.e., it calculates the interaction free energy between each water molecule and all other molecules in the simulations, avoiding double counting). Therefore, the reference state for the sum of these free energies is not the dehydrated GIST box.

## Conclusions

In this investigation, we have compared different types of simulations and free-energy methods to study water molecules in protein ligand-binding sites, in particular GCMC and GIST. We have studied two proteins: ferritin with a buried binding site and galectin-3C which binds ligands on the surface of the protein. We have made a number of interesting observations.

- GCMC/MD is better than MD for the water sampling of buried sites—it gives a faster equilibration and better agreement between simulations started from different hydration states.
- AMBER and OpenMM MD simulations sample slightly different distributions of the number of water molecules in the binding sites—OpenMM-*NVT* seems to equilibrate faster than AMBER-*NPT*.
- Crystal water molecules seem to be reasonably well reproduced by all simulations and the performance of the various methods is variable for ferritin, whereas for galectin-3C, MD seems to be better than GCMC.
- GIST and GCMC solvation free energies are not comparable because they use different reference states.
- Restraints in the MD simulations not only improve the precision of calculated GIST enthalpies and entropies but also significantly change them. There is a good correlation of the free energies of individual water molecules calculated from restrained or unrestrained simulations, but there are significant quantitative differences.

Consequently, GCMC/MD can be recommended for the study of the water structure and energetics in buried binding sites of proteins. Conventional MD seems to be preferable for a water-exposed binding site and GIST seems to give qualitatively reliable water thermodynamics, although the use of restraints may significantly affect the quantitative measures.

## Supporting Information Available

Details of the setup of the simulations; example time courses of the number of water molecules in the ferritin simulations; results of the fitting of the exponential model to the number of water molecules in the ferritin simulations; comparison of water clusters or densities from simulations and crystallographic water sites for ferritin and galectin-3C; hydrogen bonds involving water in crystal structures of galectin-3C; GCMC titration curves for both ferritin and galectin-3C; GIST energy and entropy components; and comparisons of the GIST entropy and enthalpy components between individual water sites in the restrained and unrestrained simulations of both ferritin and galectin-3C (PDF)
