# Supplementary material for: Comparison of Grand Canonical and Conventional Molecular Dynamics Simulation Methods for Protein-Bound Water Networks
Source: ACS Phys Chem Au. 2022 Feb 11;2(3):247–59. doi: 10.1021/acsphyschemau.1c00052 (PMC9136951; doi:10.1021/acsphyschemau.1c00052)
Supplement: Supplementary file 1 — pg1c00052_si_001.pdf [file pg1c00052_si_001.pdf]

Supporting Information

Comparison of Grand Canonical and Conventional Molecular Dynamics Simulation Methods for Protein-Bound Water Networks

Vilhelm Ekberg,[a] Marley L. Samways,[b] Majda Misini Ignjatović,[a] Jonathan W. Essex [b] & Ulf Ryde *[a]

[a] Department of Theoretical Chemistry, Lund University, Chemical Centre, P. O. Box 124, SE-221 00 Lund, Sweden

[b] School of Chemistry, University of Southampton, Southampton, SO17 1BJ, U.K

Correspondence to Ulf.Ryde@teokem.lu.se

2021-12-07

Additional Simulation Details

Protein Preparation

In both proteins simulated, all Glu and Asp residues were taken as negatively charged, and all Lys and Arg residues were taken as positively charged. For ferritin, the His49, His132 and His147 residues were positively charged, and the His124 and His144 residues were protonated on the NE2 atom, based on investigation of the hydrogen-bonding network and solvent accessibility,[1,2] resulting in a protein charge of –8. For galectin-3C, the His158 residue in the binding site was protonated on the ND1 atom, whereas the other three His residues were protonated on the NE2 atoms, in accordance with the neutron structure of the lactose-bound state,[3] NMR measurements and previous MD investigations,[4] resulting in a protein charge of +4. All crystallographic water molecules were retained in the simulations, except the simulations labelled as "dry", in which waters within the region of interest (ROI) were removed prior to simulation.

Ligand Parameters

Ligand geometries were optimised using the semi-empirical AM1 method,[5] followed by a single-point calculation at the HF/6-31G* level of theory to calculate the molecular electrostatic potential, sampled with the Merz–Kollman scheme.[6] These calculations were performed using Gaussian 09.[7] The charges were then determined by the restrained electrostatic potential method,[8] using antechamber.[9] For the galectin-3C ligands, a few missing parameters were assigned[10] using the Seminario approach.[11,12]

GCMC Simulations for Ferritin

In order to ensure that the water molecules present in the ROI were equilibrated prior to MD simulations, the *apo*- and *holo*-structures of ferritin were both subjected to a GCMC titration, as implemented in version 3.4 the ProtoMS software package. Prior to this, the initial structure was subjected to 100 steps of steepest-descent minimisation in AMBER 14,[13] and then solvated in a spherical droplet of TIP3P[14] water molecules (with a radius of 30 Å). These simulations were carried out at 298 K, with a cutoff of 10 Å was applied for non-bonded interactions, with a switching function applied over the last 0.5 Å.

The system was simulated at 30 equally-spaced B values, from –23.925 to –0.725 ($B_{\text{equil}} = -6.325$). In order to remove bias, any waters present in the ROI were removed prior to the simulation. Each simulation at each B value involved an initial equilibration of 5M moves of only GCMC sampling, split equally between insertions, deletions and configurational sampling of waters within the ROI. This was followed by a second equilibration of 5M moves, with GCMC sampling and configurational sampling with equal probabilities. The system was then simulated for 200M moves of production, with the same moves ratios, with the addition of replica exchanges attempted between adjacent B values, every 100k moves.

These titrations were carried out under both constrained and unconstrained conditions, where in the former, configurational sampling was applied only to water molecules, and in the latter, configurational sampling was also applied to the protein and ligand. Three independent repeats were carried out under each set of conditions, for both the *holo*- and *apo*-structures.

Construction of a Starting Structure for Molecular Dynamics of Ferritin

For both ferritin structures, a simulation frame was taken from a constrained GCMC simulation replica at B_{equil} and then solvated in a cuboidal ROI, with sodium ions added to neutralise the system charge. The volume of the system was equilibrated for 1 ns at constant temperature and pressure, with all protein atoms restrained to their initial positions, using harmonic restraints with a force constant of 10 kcal mol^{-1} Å^{-2}. These simulations were carried out in OpenMM,

under the same conditions as the other OpenMM simulations described below, except for the addition of the harmonic restraints, and a Monte Carlo barostat (volume changes attempted every 25 timesteps).

After this, the protein positions were reset to their original values, as were the water sites present within the ROI (9 waters for the *apo*-structure and 4 for the *holo*-structure). These structures were used to run all MD simulations referred to as "wet". Copies of these structures were also created, with the waters within the ROI removed, and these were used as the initial structure for all simulations labelled as "dry".

OpenMM Simulations for Ferritin

These simulations were all carried out using version 7.2.2 of OpenMM,[15] with a non-bonded cutoff of 12 Å, with a switching function applied over the last 2 Å, and long-range electrostatic interactions calculated using the particle mesh Ewald (PME) method.[16] Dynamics were integrated at a temperature of 298 K, using the BAOAB Langevin integrator with a timestep of 2 fs and a friction coefficient of 1 ps^{-1}. All bonds involving hydrogen atoms were constrained using the SETTLE algorithm for water and the SHAKE algorithm, otherwise. These simulations were run at constant volume for 25 ns, with simulation frames saved every 12.5 ps.

Two sets of calculations were run: the constrained simulations involved constraints applied to the protein and ligand atoms (towards the starting crystal structure), in order to ensure that they did not move, but under the unconstrained simulations, they were able to move freely.

An equivalent set of simulations were carried out using GCMC/MD, via the *grand* Python module (version 1.0.0).[17] These simulations were carried out under identical conditions to the canonical simulations described above, with the addition that 100 GCMC insertions/deletions of water molecules were attempted for every 2.5 ps of MD. These moves were applied to a spherical region, centred on the mean coordinate of the C_α atoms of the two Tyr27 residues, with a radius of 8.5 Å. Three independent repeats were run for each of the sixteen types of simulations (MD or GCMC/MD, *apo* or *holo,* wet or dry, constrained or unconstrained).

AMBER Simulations for Ferritin

These simulations were performed in AMBER 16,[9] using a non-bonded cutoff of 10 Å, with the PME method used to calculate the effects of long-range electrostatic interactions.[16] The temperature of the system was maintained at 298 K, using a Langevin thermostat with a collision frequency of 2 ps^{-1},[18] and the pressure was maintained at 1.0 bar using the Berendsen barostat with a relaxation time of 1.0 ps.[19] A timestep of 2 fs was used, with all bonds involving hydrogen atoms constrained using the SHAKE algorithm. These simulations were equilibrated for 1 ns at constant pressure, followed by 25 ns of constant pressure production, with simulation frames saved every 10 ps.

Two sets of calculations were performed for each type. In the restrained simulations, protein and ligand heavy atoms were restrained to their initial positions, using harmonic restraints with a force constant of 10 kcal mol^{-1} $Å^{-2}$. The unrestrained simulations involved no such restraints. Ten independent repeats were performed for each of the eight types of simulations of ferritin (*apo* or *holo,* wet or dry, restrained or unrestrained).

GCMC Simulations for Galectin-3C

The GCMC simulations for the galectin-3C systems were carried out under almost identical conditions to the titrations described above for ferritin – as such, only differences are described here. The protein structure was minimised in the same way as before, but was then solvated in

a droplet of TIP4P water molecules[14] with a radius of 30 Å. The system was simulated at a temperature of 300 K, with 111 evenly spaced B values, from –29.770 to –2.270 ($B_{\text{equil}} = -5.270$). The equilibration and production sections of the simulation were executed identically. Three independent sets of titrations were run under both constrained and unconstrained conditions for both diastereomers.

AMBER Simulations for Galectin-3C

These simulations were performed in AMBER 14,[13] under four different sets of conditions, in order to investigate their impact on the results obtained from a GIST analysis. These simulations are denoted C, U, R3 and R. In the C (constrained) simulations, the protein and ligand atoms were constrained to their crystallographic positions. In the U (unrestrained) simulation, the protein and ligand were allowed to move freely. In the R (restrained) simulations, the protein and ligand atoms were harmonically restrained to their crystallographic positions using a force constant of 10 kcal mol^{-1} Å^{-2}.

The R3 results were taken from a previous study.[10] Here, the U simulations were clustered with the *cluster* command of the cpptraj module[20] with respect to the conformation of the ligand. Representative structures were then selected for the three largest clusters for the R ligand (with weights 0.8, 0.1 and 0.1) and the four largest clusters for the S ligand (the S ligand shows two conformations in the crystal structure, and we took two clusters of each, with final weights of 0.47, 0.09, 0.37 and 0.06). Simulations were carried out in which the ligand was restrained to each of these representative structures, and the protein was restrained to the crystal structure, using the same force constant as for R.[10]

All bonds involving hydrogen atoms were constrained using the SHAKE algorithm.[21] No neutralising ions were added in any case – where PME calculations were performed, a continuous, neutralising charge distribution was employed. The C simulations were first minimised for 100 steps of minimisation, then equilibrated for 1 ns of constant volume MD, before running 10 ns of constant volume production. The other simulations were first minimised for 1000 steps of minimisation, then equilibrated for 20 ps of constant volume MD and 1 ns of constant pressure MD, before running 10 ns of constant pressure production. For all simulations, frames were saved every 1 ps.

For the C simulations, the protein was solvated in a non-periodic spherical droplet of TIP4P water,[14] with a radius of 33 Å, whereas in the other simulations, it was solvated in a periodic, truncated octahedral box of TIP4P-Ew water.[22] The performance of the various water models depend on what property is studied. TIP4P-Ew gives a better reproduction of bulk water structural and dynamic properties, whereas TIP3P typically give better hydration free energies.[23,24] In both cases, the solvent was made sure to extend at least 10 Å from the protein. A nonbonded cutoff of 10 Å was used for the C simulations, and a value of 8 Å was used for the other simulations (these calculated long-range electrostatic interactions using PME[16]). The temperature was maintained using Berendsen's weak-coupling algorithm[19] for the C simulations, and using a Langevin thermostat (with a collision frequency of 2 ps^{-1}) for the other simulations.

The C & R simulations were analysed using a ROI with dimensions of 27.0 Å × 13.5 Å × 15.0 Å, whereas the dimensions used for the U & R3 simulations were 30.0 Å × 21.0 Å × 21.0 Å, owing to the greater movement of the ligand (the ROIs were selected to always include all conformations of the ligands). Ten independent repeats were carried out for each set of simulations (resulting in 30–40 repeats per ligand for the R3 simulations).

Figure S1. Representative time courses for the number of water molecules in ferritin, demonstrating the fitting of the exponential model for the equilibration. Shown here are the graphs for the AUD-NVT (left) and the HUW-NVT (right) simulations. The fit is shown as the solid black line, the mean value of N (over all independent repeats) at each point in time is shown as the solid blue line, and the shaded region represents one standard deviation either side of the mean.

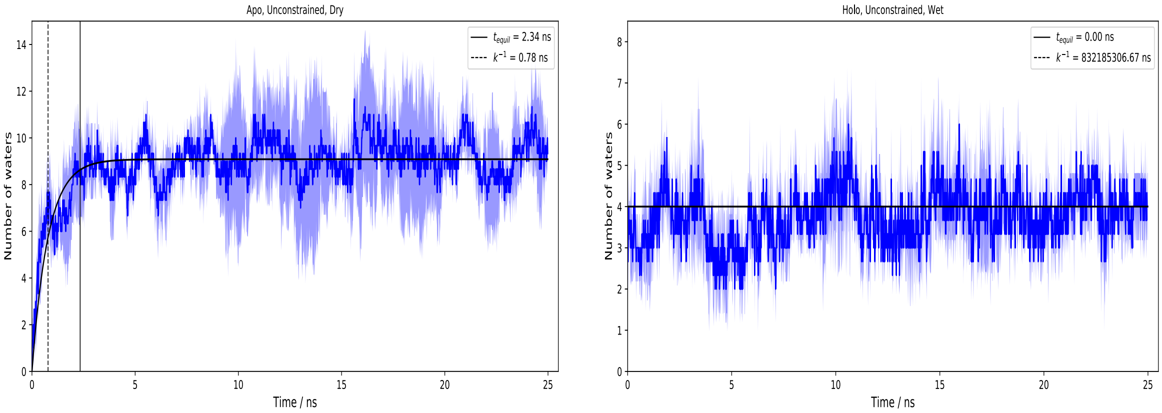

Table S1. Results from the fitting of the exponential model to the number of water molecules in different ferritin simulations, along with the equilibrated mean number of waters in the ROI. SD is the standard deviation of the mean value of N (averaged over independent repeats for each point in time). The standard error (SE) is determined by calculating the mean value of N separately for each repeat (after the equilibration time), and then calculating the standard error of the mean over these values. Values of a reported as integers were constrained during the fit.

Simulation	Ensemble	a	b	k / ns^{-1}	t_{eq} / ns	$\langle N \rangle_{eq}$	SD	SE
ARD	NPT	0	7.5	4.3	12.8	7.4	1.6	0.4
ARW	NPT	8.0	0.0	0.0	0.0	8.0	1.6	0.3
AUD	NPT	0	8.8	1.0	2.9	8.7	2.0	0.3
AUW	NPT	9	0.0	0.0	0.0	8.7	1.8	0.1
HRD	NPT	0	3.2	1.4	4.1	3.2	0.8	0.1
HRW	NPT	3.6	0.0	0.0	0.0	3.6	0.7	0.1
HUD	NPT	0	3.8	0.9	2.6	3.8	1.2	0.1
HUW	NPT	4	0.0	0.0	0.0	3.8	1.3	0.1
ACD	NVT	0	7.3	0.4	7.1	7.3	1.2	0.4
ACW	NVT	9	0.0	0.0	0.0	8.6	0.9	0.3
AUD	NVT	0	9.1	1.3	2.3	9.1	2.1	0.6
AUW	NVT	9	0.0	0.0	0.0	8.3	2.0	0.3
HCD	NVT	0	2.9	25.8	0.1	2.9	0.8	0.3
HCW	NVT	4	0.0	0.0	0.0	3.8	0.5	0.1
HUD	NVT	0	4.4	2.6	1.2	4.4	1.2	0.2
HUW	NVT	4	0.0	0.0	0.0	3.8	1.1	0.1
ACD	μVT	0	8.1	2.6	1.1	8.1	1.6	0.1
ACW	μVT	9	0.0	0.0	0.0	8.2	1.6	0.3
AUD	μVT	0	8.2	5.4	0.6	8.2	1.8	0.2
AUW	μVT	9	0.0	1.3	0.0	8.3	2.1	0.1
HCD	μVT	0	3.2	20.6	0.1	3.2	0.7	0.1
HCW	μVT	4	0.0	0.0	0.0	3.0	0.7	0.0
HUD	μVT	0	3.8	4.7	0.6	3.8	1.4	0.5
HUW	μVT	4	0.0	0.0	0.0	3.6	1.0	0.0

Figure S2. Representative images showing the comparison between water clusters extracted from the ferritin simulations, and the crystallographic water sites. The crystallographic sites are shown in grey, and the simulated sites are shown in red, with the intensity of the colour scaled according to their occupancy (only clusters present for at least 30% of the simulation are shown). The left-hand side shows the clusters for one of the AUW-NPT-AMBER repeats, where three of the four crystallographic sites are matched to within 2.0 Å. The right-hand side shows the comparison for one of the HCD-μVT-OpenMM repeats, where both crystallographic sites are reproduced within 0.7 Å.

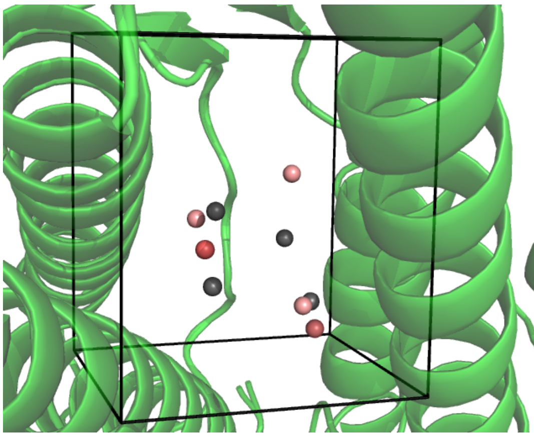

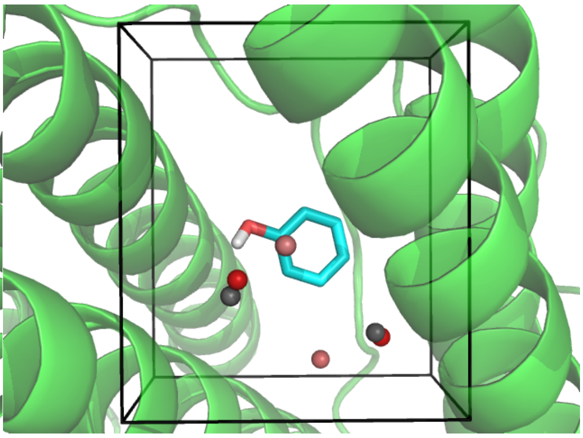

Figure S3. Comparison between water probability densities from the Amber MD (grey), the OpenMM MD (green) and the GCMC/MD (magenta) simulations of ferritin, shown for the ARW, AUD, AUW, ARD, HRW, HUD, HUW and HRD simulations. Densities are shown for an isovalue of 0.3. The crystallographic water molecules are shown as balls, coloured according to their temperature factors (scale shown at the bottom of the figure).

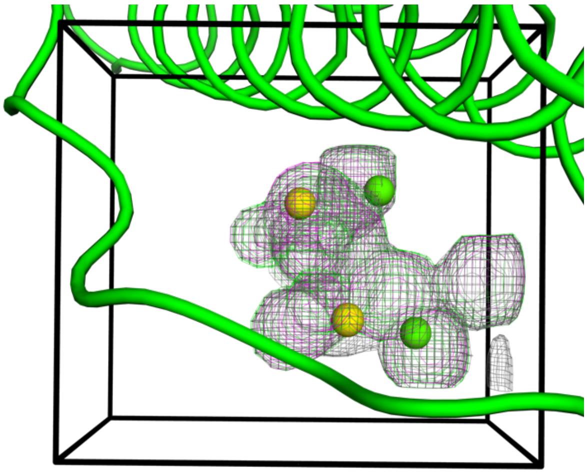

ARW

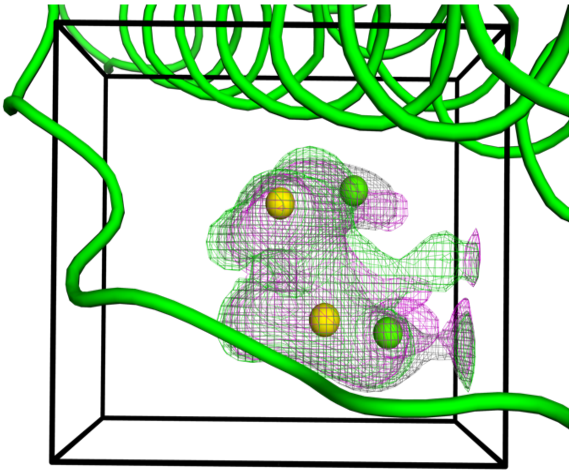

AUD

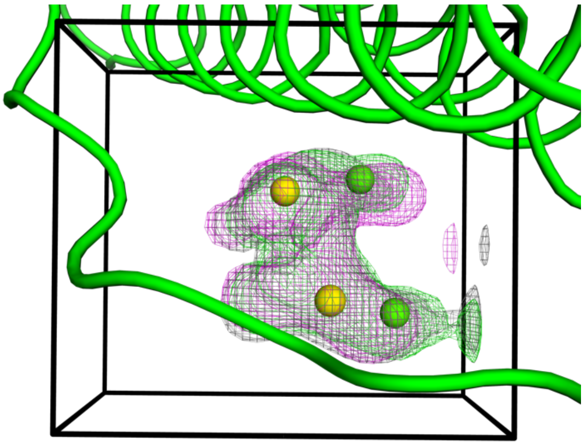

AUW

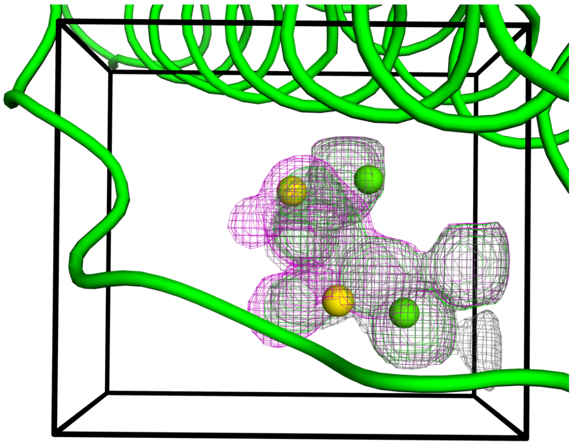

ARD

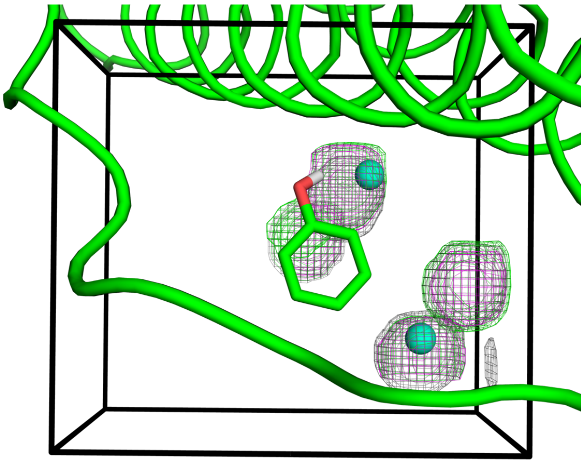

HRW

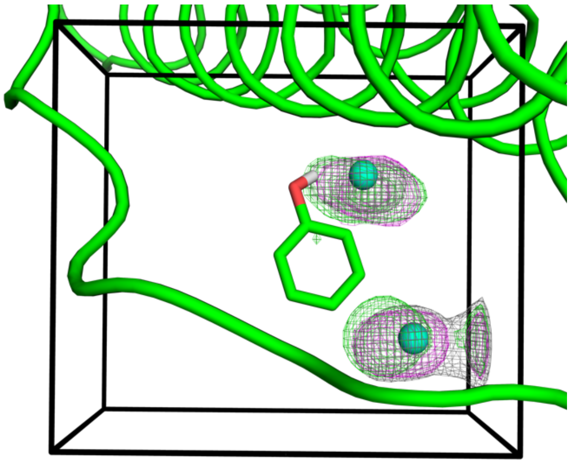

HUD

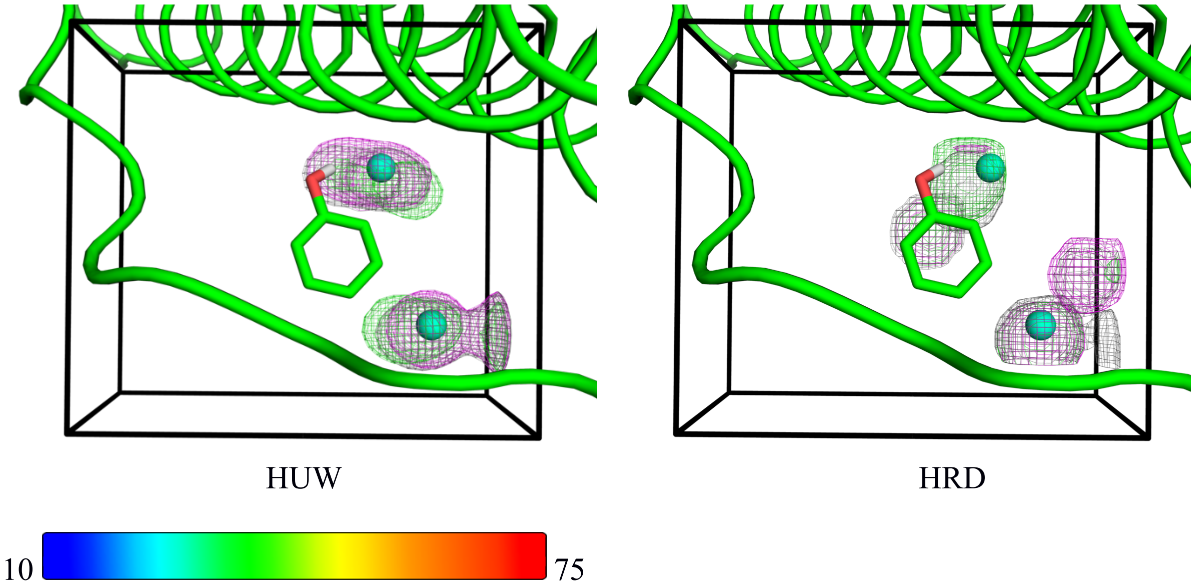
HUW
HRD
10
75

Figure S4. Density maps comparing the water sampling observed using constrained MD (AMBER, blue) or GCMC (ProtoMS, magenta) for the S ligand in complex with galectin-3C. The protein and ligand are shown with the crystallographic coordinates, and the experimental water sites are coloured according to their temperature factors (scale shown at the bottom of the figure). Water molecules that make hydrogen bonds with the protein or the ligand (Table S2) are marked with residue numbers. The density maps are contoured at an isovalue of 0.6.

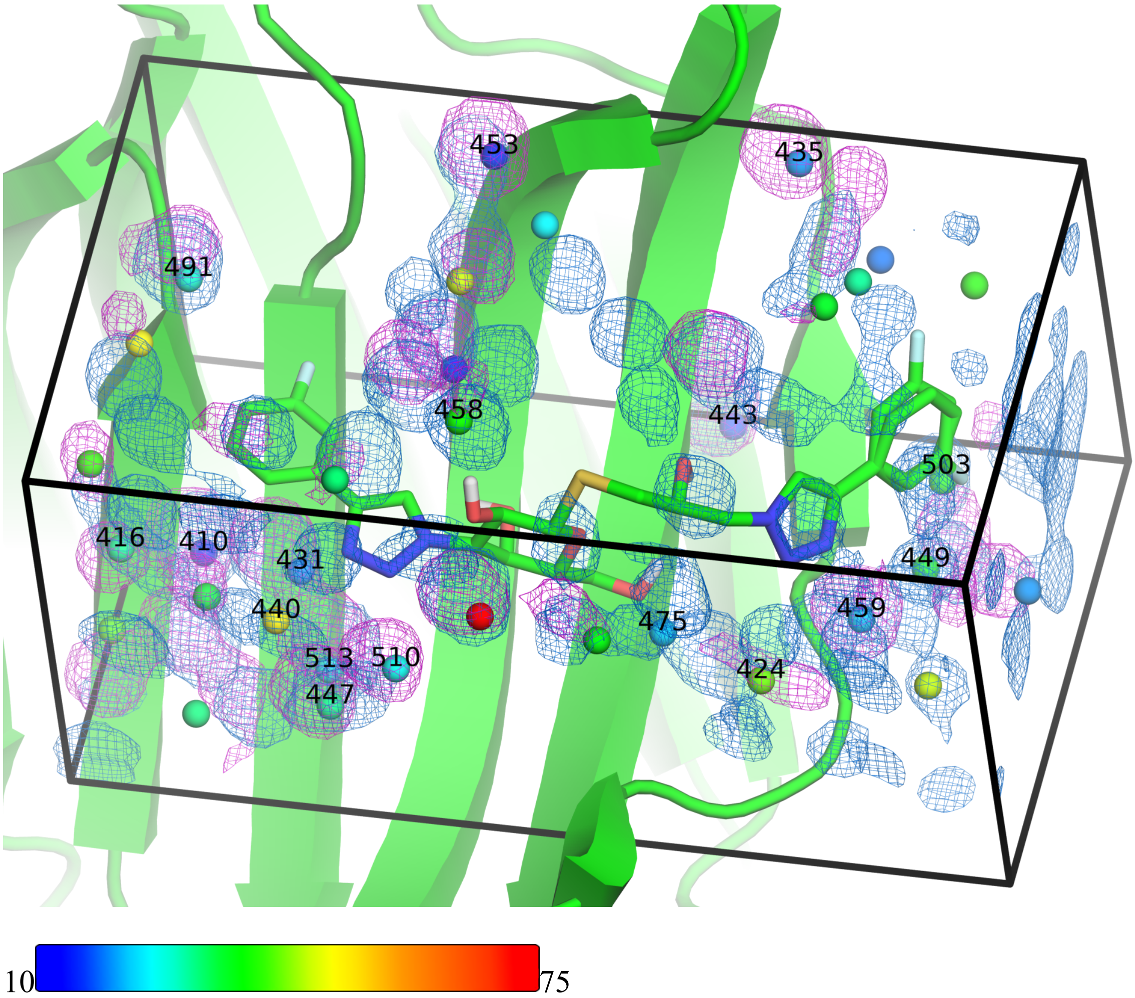

Table S2. Hydrogen-bond interactions between the crystal-water molecules inside the ROI of galectin-3C and the protein or the ligand. The distances between the O atoms of the water molecules and the protein and ligand N or O atoms are shown (in Å). Only distances smaller than 3.0 Å were included. The residue numbers are those in the 6QGF and 6QGE crystal structures.[10] Water molecule 435 for the S ligand is located slightly outside of the ROI, but is included, because it is enclosed by GCMC probability density in Figure S3.

R			S		
Wat	Atom	Dist.	Wat	Atom	Dist.
407	OD2 Asp148	2.6	410	OD2 Asp148	2.6
407	OG Ser237	2.7	410	OG Ser237	2.8
407	OG Ser237	2.6	410	OG Ser237	2.6
419	O Glu165	2.8	416	OG Ser237	2.6
419	OE1 Glu165	2.7	424	O Gly182	2.7
421	OD1 Asp239	2.7	431	OD2 Asp148	2.7
425	O Ile171	3.0	435	O Glu165	2.8
425	O Glu185	2.7	435	OE1 Glu165	2.7
426	OD2 Asp148	2.7	440	N01 S	2.7
428	O22 R	2.7	443	O Ile171	2.8
450	NZ Lys176	2.8	443	O Glu185	2.7
457	O Asn143	2.8	447	NZ Lys176	2.7
457	O Asn143	2.8	449	O Glu184	2.7
457	N Asn164	2.9	453	O Asn143	2.8
465	N Arg186	2.8	453	O Asn143	2.8
470	NZ Lys233	2.8	453	N Asn164	2.9
474	OE2 Glu165	2.9	458	O04 S	2.8
481	OG Ser237	2.9	459	N Glu184	2.9
484	NE1 Trp181	2.9	459	N06 S	2.8
485	O Gly182	2.9	475	O03 S	2.8
493	OG Ser237	2.9	491	NE Arg144	2.9
496	O21 R	2.9	491	OD1 Asp239	3.0
498	O22 R	2.9	503	N Arg186	2.9
505	OD1 Asp148	3.0	510	NE1 Trp181	2.9
512	NH1 Arg144	3.0	510	N02 S	3.0
			513	OD1 Asp148	3.0

Figure S5. GCMC titration plots for *apo*-ferritin. On the left are the titration curves, and on the right are the resulting plots of the water network binding free energy, as a function of the number of waters. The upper row represents the constrained simulations, and the lower row represents the unconstrained simulations.

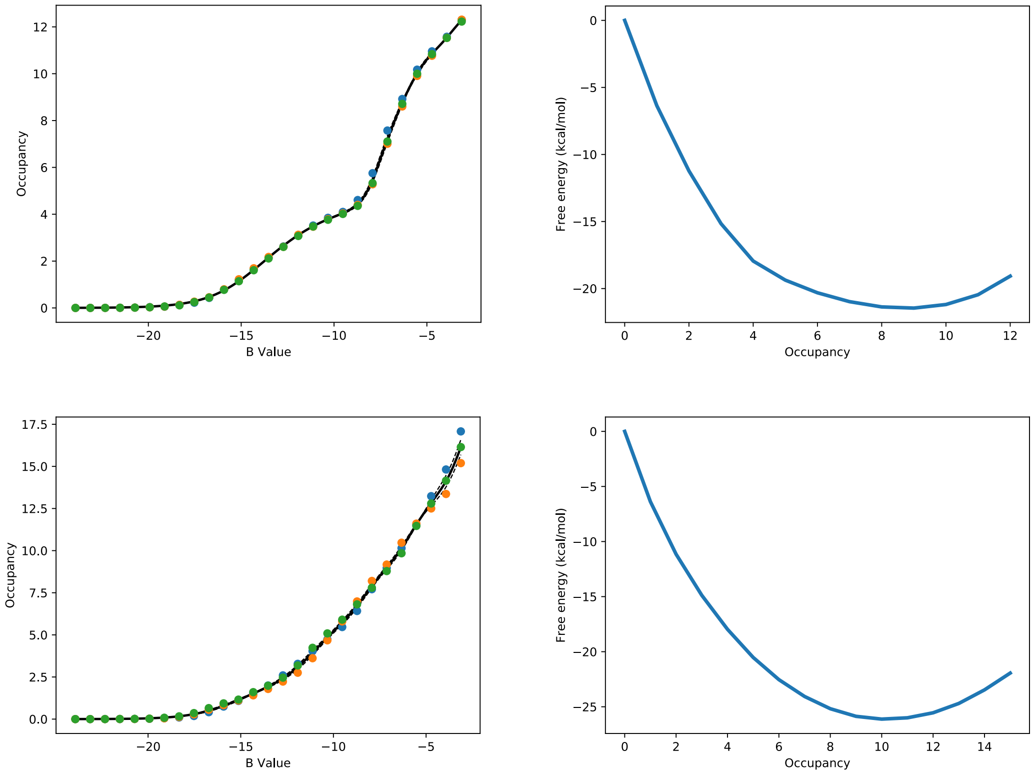

Figure S6. GCMC titration plots for *holo*-ferritin. On the left are the titration curves, and on the right are the resulting plots of the water network binding free energy, as a function of the number of waters. The upper row represents the constrained simulations, and the lower row represents the unconstrained simulations.

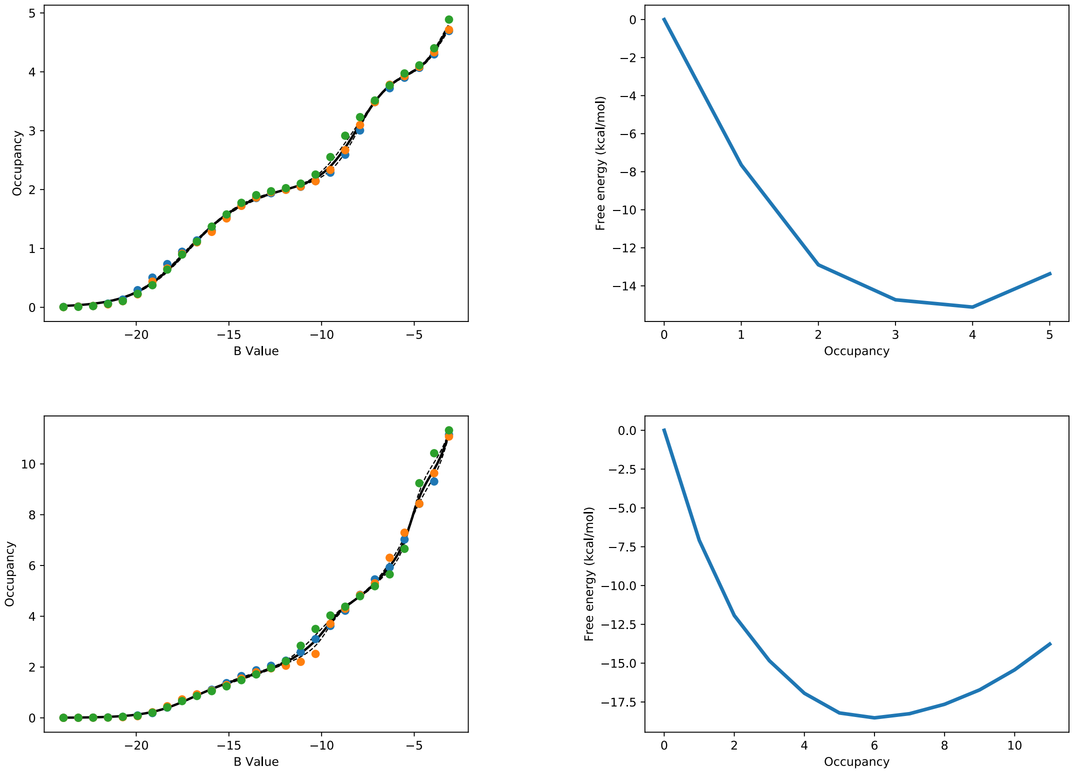

Figure S7. GCMC titration plots for galectin-3C with the R ligand. On the left are the titration curves, and on the right are the resulting plots of the water network binding free energy, as a function of the number of waters. The upper row represents the constrained simulations, and the lower row represents the unconstrained simulations.

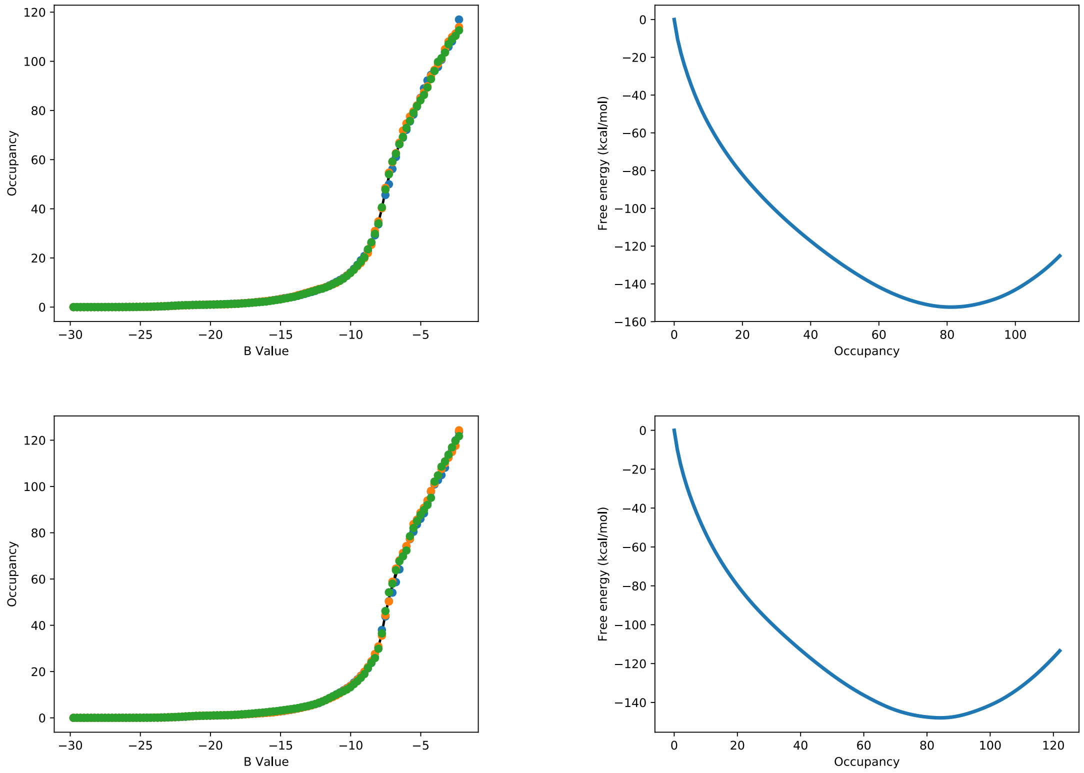

Figure S8. GCMC titration plots for galectin-3C with the S ligand. On the left are the titration curves, and on the right are the resulting plots of the water network binding free energy, as a function of the number of waters. The upper row represents the constrained simulations, and the lower row represents the unconstrained simulations.

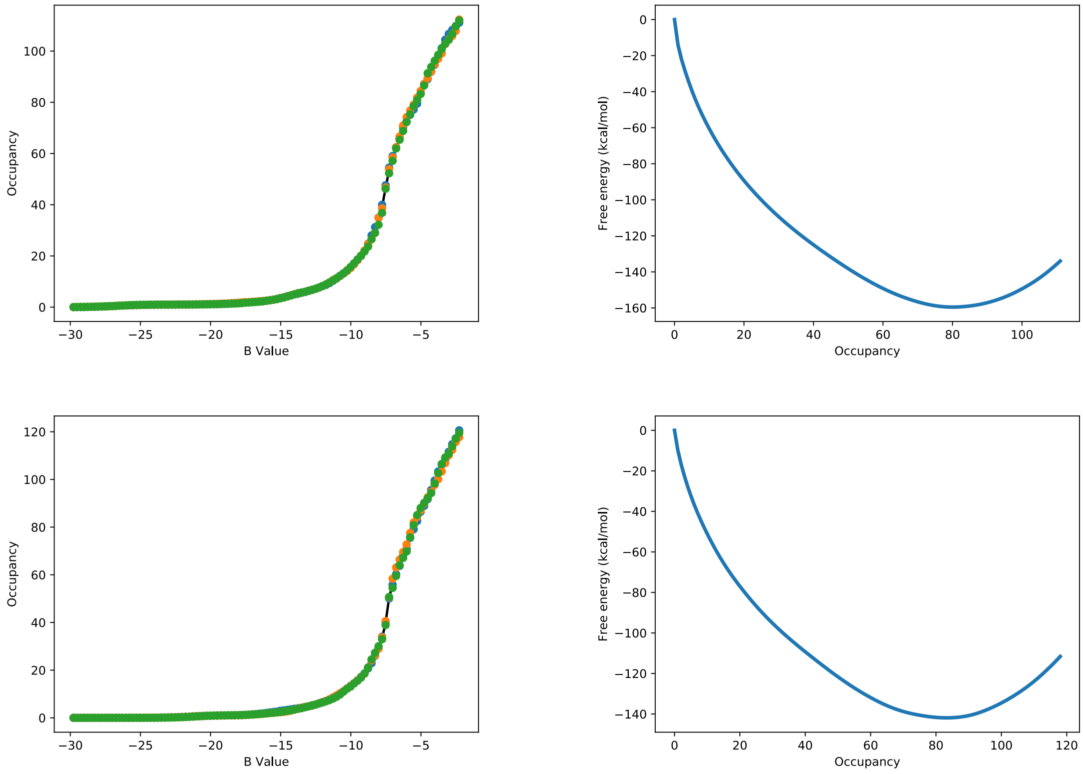

Table S3. Individual energy and entropy components (kJ/mol) from the GIST analysis of the AMBER simulations of ferritin.

System	$\Delta U_{\text{sw}}^{\text{ROI}}$	$\Delta U_{\text{ww}}^{\text{ROI}}$	$-T\Delta S_{\text{sw}}^{\text{ROI,trans}}$	$-T\Delta S_{\text{sw}}^{\text{ROI,orient}}$
ARD	–249.2 ± 5.0	–121.1 ± 8.3	26.7 ± 1.2	39.2 ± 1.3
ARW	–279.5 ± 5.6	–167.3 ± 10.7	36.6 ± 2.6	49.3 ± 2.3
AUD	–323.2 ± 15.3	–181.0 ± 6.8	10.8 ± 1.2	34.3 ± 2.2
AUW	–286.3 ± 12.0	–190.2 ± 4.6	11.2 ± 1.3	32.6 ± 2.3
HRD	–195.6 ± 4.3	–19.2 ± 0.7	22.1 ± 0.8	26.3 ± 0.4
HRW	–215.5 ± 2.7	–19.4 ± 0.6	25.5 ± 0.6	28.0 ± 0.4
HUD	–213.4 ± 8.8	–49.1 ± 4.1	6.0 ± 0.8	20.8 ± 1.2
HUW	–215.2 ± 8.1	–48.7 ± 3.7	6.6 ± 0.3	21.0 ± 0.7

Table S4. Comparison of the GIST entropy and enthalpy components between individual water sites in the restrained and unrestrained simulations of ferritin and galectin-3C (corresponding to the free energies in Table 6). The first part of the table gives the correlation coefficient (*R*), the second part the mean absolute deviation (MAD in kJ/mol).

	R					MAD				
	$-T\Delta S_{\text{tr}}$	$-T\Delta S_{\text{rot}}$	ΔE_{sw}	ΔE_{ww}	ΔG_{tot}	$-T\Delta S_{\text{tr}}$	$-T\Delta S_{\text{rot}}$	ΔE_{sw}	ΔE_{ww}	ΔG_{tot}
ARD–AUD	0.28	0.62	0.76	0.44	0.89	2.4	2.7	8.0	5.4	5.4
ARW–AUW	0.35	0.64	0.86	0.57	0.84	2.5	2.6	7.8	4.6	8.0
HRD–HUD	0.56	0.49	0.35	-0.73	0.59	4.3	3.9	17.0	12.8	11.4
HRW–HUW	0.59	0.40	0.11	-0.67	0.64	4.9	4.0	16.9	8.9	10.7
RR–RC	0.56	0.82	0.91	0.87	0.85	3.2	1.2	4.4	6.4	6.4
SR–SC	0.59	0.87	0.96	0.93	0.91	3.0	1.2	3.9	6.4	6.9
RU–RR3	0.68	0.80	0.89	0.87	0.88	1.1	1.1	3.1	2.1	3.3
SU–SR3	0.60	0.79	0.83	0.83	0.79	1.2	1.1	3.7	2.4	3.6
RU–RR3[a]	0.52	0.74	0.87	0.82	0.82	1.4	1.3	3.6	2.5	3.9
SU–SR3[a]	0.54	0.75	0.86	0.82	0.84	1.4	1.2	3.7	2.7	3.9
SU–SR3[b]	0.59	0.76	0.87	0.84	0.86	1.4	1.2	3.6	2.6	3.6

[a] Using the second largest cluster for the R3 simulation (with 684 and 678 water molecules for R and S, respectively).

[b] Using the largest cluster for the second conformation of the S ligand for the R3 simulation (with 735 water molecules).

Figure S9. Examples of the correlation between the GIST Δ*G* for different water sites calculated from restrained and unrestrained simulations of ferritin (top) and galectin-3C (bottom).

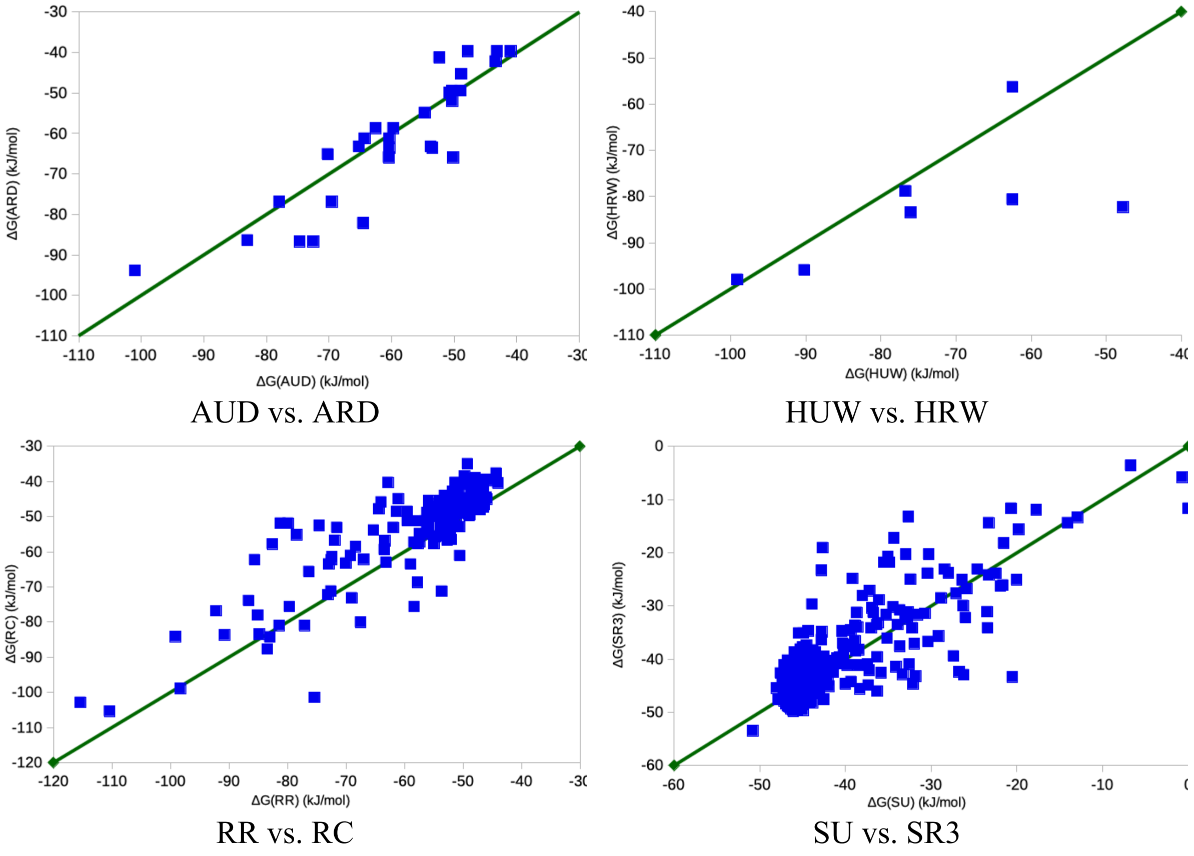

Table S5. Breakdown of the thermodynamic parameters calculated from the GIST analysis of the AMBER simulations of galectin-3C. All values are given in units of kJ/mol.

Ligand	Simulation	$\Delta U_{\mathrm{sw}}^{\mathrm{ROI}}$	$\Delta U_{\mathrm{ww}}^{\mathrm{ROI}}$	$-T\Delta S_{\mathrm{sw}}^{\mathrm{ROI,trans}}$	$-T\Delta S_{\mathrm{sw}}^{\mathrm{ROI,orient}}$
R	C	–1466 ± 4	–2975 ± 5	411 ± 2	322 ± 2
	R	–1722 ± 1	–3222 ± 2	183.3 ± 0.4	225.2 ± 0.6
	R3	–2914 ± 2	–12814 ± 2	319.3 ± 0.4	398.8 ± 0.6
	U	–2794 ± 24	–12316 ± 17	91 ± 4	–262 ± 4
S	C	–1483 ± 6	–2962 ± 7	396 ± 3	327 ± 3
	R	–1644 ± 1	–3226 ± 1	187.1 ± 0.3	218.3 ± 0.5
	R3	–2805 ± 1	–12877 ± 2	317.4 ± 0.3	397.7 ± 1.3
	U	–2906 ± 33	–12198 ± 33	94 ± 3	–252 ± 5

References

(1) Genheden, S.; Mikulskis, P.; Hu, L.; Kongsted, J.; Söderhjelm, P.; Ryde, U. Accurate Predictions of Nonpolar Solvation Free Energies Require Explicit Consideration of Binding-Site Hydration. *J. Am. Chem. Soc.* **2011**, *133*, 13081–13092. https://doi.org/10.1021/ja202972m.

(2) Mikulskis, P.; Genheden, S.; Wichmann, K.; Ryde, U. A Semiempirical Approach to Ligand-Binding Affinities: Dependence on the Hamiltonian and Corrections. *J. Comput. Chem.* **2012**, *33*, 1179–1189. https://doi.org/https://doi.org/10.1002/jcc.22949.

(3) Manzoni, F.; Wallerstein, J.; Schrader, T. E.; Ostermann, A.; Coates, L.; Akke, M.; Blakeley, M. P.; Oksanen, E.; Logan, D. T. Elucidation of Hydrogen Bonding Patterns in Ligand-Free, Lactose- and Glycerol-Bound Galectin-3C by Neutron Crystallography to Guide Drug Design. *J. Med. Chem.* **2018**, *61*, 4412–4420. https://doi.org/10.1021/acs.jmedchem.8b00081.

(4) Uranga, J.; Mikulskis, P.; Genheden, S.; Ryde, U. Can the Protonation State of Histidine Residues Be Determined from Molecular Dynamics Simulations? *Comput. Theor. Chem.* **2012**, *1000*, 75–84. https://doi.org/10.1016/j.comptc.2012.09.025.

(5) Dewar, M. J. S.; Zoebisch, E. G.; Healy, E. F.; Stewart, J. J. P. Development and Use of Quantum Mechanical Molecular Models. 76. AM1: A New General Purpose Quantum Mechanical Molecular Model. *J. Am. Chem. Soc.* **1985**, *107*, 3902–3909. https://doi.org/10.1021/ja00299a024.

(6) Besler, B. H.; Merz, K. M.; Kollman, P. A. Atomic Charges Derived from Semiempirical Methods. *J. Comput. Chem.* **1990**, *11* (4), 431–439. https://doi.org/10.1002/jcc.540110404.

(7) Frisch, M. J.; Trucks, G. W.; Schlegel, H. B.; Scuseria, G. E.; Robb, M. A.; Cheeseman, J. R.; Scalmani, G.; Barone, V.; Mennucci, B.; Petersson, G. A.; Nakatsuji, H.; Caricato, M.; Li, X.; Hratchian, H. P.; Izmaylov, A. F.; Bloino, J.; Zheng, G.; Sonnenberg, J. L.; Hada, M.; Ehara, M.; Toyota, K.; Fukuda, R.; Hasegawa, J.; Ishida, M.; Nakajima, T.; Honda, Y.; Kitao, O.; Nakai, H.; Vreven, T.; Montgomery, Jr., J. A.; Peralta, J. E.; Ogliaro, F.; Bearpark, M.; Heyd, J. J.; Brothers, E.; Kudin, K. N.; Staroverov, V. N.; Kobayashi, R.; Normand, J.; Raghavachari, K.; Rendell, A.; Burant, J. C.; Iyengar, S. S.; Tomasi, J.; Cossi, M.; Rega, N.; Millam, N. J.; Klene, M.; Knox, J. E.; Cross, J. B.; Bakken, V.; Adamo, C.; Jaramillo, J.; Gomperts, R.; Stratmann, R. E.; Yazyev, O.; Austin, A. J.; Cammi, R.; Pomelli, C.; Ochterski, J. W.; Martin, R. L.; Morokuma, K.; Zakrzewski, V. G.; Voth, G. A.; Salvador, P.; Dannenberg, J. J.; Dapprich, S.; Daniels, A. D.; Farkas, Ö.; Foresman, J. B.; Ortiz, J. V. .; Cioslowski, J.; Fox, D. J. Gaussian09. *Journal of the American Statistical Association*. 2009. https://doi.org/10.1198/jasa.2001.s400.

(8) Bayly, C. I.; Cieplak, P.; Cornell, W. D.; Kollman, P. A. A Well-Behaved Electrostatic Potential Based Method Using Charge Restraints for Deriving Atomic Charges: The RESP Model. *J. Phys. Chem.* **1993**, *97* (40), 10269–10280. https://doi.org/10.1021/j100142a004.

(9) Case, D. A.; Cerutti, D. S.; Cheatham, III, T. E.; Darden, T. A.; Duke, R. E.; Giese, T. J.; Gohlke, H.; Goetz, A. W.; Greene, D.; Homeyer, N.; Izadi, S.; Kovalenko, A.; Lee, T. S.; LeGrand, S.; Li, P.; Lin, C.; Liu, J.; Luchko, T.; Luo, R.; Mermelstein, D.; Merz, K. M.; Monard, G.; Nguyen, H.; Omelyan, I. P.; Onufriev, A. V.; Pan, F.; Qi, R.; Roe, D. R.; Roitberg, A. E.; Sagui, C.; Simmerling, C. L.; Botello-Smith, W. M.; Swails, J.; Walker, R. C.; Wang, J.; Wolf, R. M.; Wu, X.; Xiao, L.; York, D. M.; Kollman, P. A. Amber 16. University of California, San Francisco 2017.

(10) Verteramo, M. L.; Stenström, O.; Ignjatović, M. M.; Caldararu, O.; Olsson, M. A.;

Manzoni, F.; Leffler, H.; Oksanen, E.; Logan, D. T.; Nilsson, U. J.; Ryde, U.; Akke, M. Interplay between Conformational Entropy and Solvation Entropy in Protein-Ligand Binding. *J. Am. Chem. Soc.* **2019**, *141*, 2012–2026. https://doi.org/10.1021/jacs.8b11099.

(11) Seminario, J. M. Calculation of Intramolecular Force Fields from Second-Derivative Tensors. *Int. J. Quantum Chem.* **1996**, *60*, 1271–1277. https://doi.org/10.1002/(SICI)1097-461X(1996)60:7<1271::AID-QUA8>3.0.CO;2-W.

(12) Nilsson, K.; Lecerof, D.; Sigfridsson, E.; Ryde, U. An Automatic Method to Generate Force-Field Parameters for Hetero-Compounds. *Acta Crystallogr. - Sect. D Biol. Crystallogr.* **2003**, *59*, 274–289. https://doi.org/10.1107/S0907444902021431.

(13) Case, D. A.; Berryman, J. T.; Betz, R. M.; Cerutti, D. S.; Cheatham, T. E.; Darden, T. A.; Duke, R. E.; Giese, T. J.; Gohlke, H.; Goetz, A. W.; Homeyer, N.; Izadi, S.; Janowski, P.; Kaus, J.; Kovalenko, A.; Lee, T. S.; LeGrand, S.; Li, P.; Luchko, T.; Luo, R.; Madej, B.; Merz, K. M.; Monard, G.; Needham, P.; Nguyen, H.; Nguyen, H. T.; Omelyan, I.; Onufriev, A.; Roe, D. R.; Roitberg, A. E.; Salomon-Ferrer, R.; Simmerling, C.; Smith, W.; Swails, J.; Walker, R. C.; Wang, J.; Wolf, R. M.; Wu, X.; York, D. M.; Kollman, P. A. AMBER 14. University of California: San Francisco 2014.

(14) Jorgensen, W. L.; Chandrasekhar, J.; Madura, J. D.; Impey, R. W.; Klein, M. L. Comparison of Simple Potential Functions for Simulating Liquid Water. *J. Chem. Phys.* **1983**, *79* (2), 926–935. https://doi.org/10.1063/1.445869.

(15) Eastman, P.; Swails, J.; Chodera, J. D.; McGibbon, R. T.; Zhao, Y.; Beauchamp, K. A.; Wang, L.-P.; Simmonett, A. C.; Harrigan, M. P.; Stern, C. D.; Wiewiora, R. P.; Brooks, B. R.; Pande, V. S. OpenMM 7: Rapid Development of High Performance Algorithms for Molecular Dynamics. *PLOS Comput. Biol.* **2017**, *13* (7), e1005659.

(16) Darden, T.; York, D.; Pedersen, L. Particle Mesh Ewald: An N·log(N) Method for Ewald Sums in Large Systems. *J. Chem. Phys.* **1993**, *98* (12), 10089. https://doi.org/10.1063/1.464397.

(17) Samways, M. L.; Bruce Macdonald, H. E.; Essex, J. W. Grand: A Python Module for Grand Canonical Water Sampling in OpenMM. *J. Chem. Inf. Model.* **2020**, *60*, 4436–4441. https://doi.org/10.1021/acs.jcim.0c00648.

(18) Wu, X.; Brooks, B. R. Self-Guided Langevin Dynamics Simulation Method. *Chem. Phys. Lett.* **2003**, *381* (3–4), 512–518. https://doi.org/10.1016/j.cplett.2003.10.013.

(19) Berendsen, H. J. C.; Postma, J. P. M.; Van Gunsteren, W. F.; DiNola, A.; Haak, J. R. Molecular Dynamics with Coupling to an External Bath. *J. Chem. Phys.* **1984**, *81* (8), 3684–3690. https://doi.org/10.1063/1.448118.

(20) Roe, D. R.; Cheatham III, T. E. PTRAJ and CPPTRAJ: Software for Processing and Analysis of Molecular Synamics Trajectory Data. *J Chem Theory Com* **2013**, *9* (7), 3084–3095. https://doi.org/10.1021/ct400341p.

(21) Ryckaert, J. P.; Ciccotti, G.; Berendsen, H. J. C. Numerical Integration of the Cartesian Equations of Motion of a System with Constraints: Molecular Dynamics of n-Alkanes. *J. Comput. Phys.* **1977**, *23* (3), 327–341. https://doi.org/10.1016/0021-9991(77)90098-5.

(22) Horn, H. W.; Swope, W. C.; Pitera, J. W.; Madura, J. D.; Dick, T. J.; Hura, G. L.; Head-Gordon, T. Development of an Improved Four-Site Water Model for Biomolecular Simulations: TIP4P-Ew. *J. Chem. Phys.* **2004**, *120* (20), 9665–9678. https://doi.org/10.1063/1.1683075.

(23) Zhang, H.; Jiang, Y.; Cui, Z.; Yin, C. Force Field Benchmark of Amino Acids. 2. Partition Coefficients between Water and Organic Solvents. *J. Chem. Inf. Model.* **2018**, *58*, 1669–1681. https://doi.org/10.1021/acs.jcim.8b00493.

(24) Kadaoluwa Pathirannahalage, S. P.; Meftahi, N.; Elbourne, A.; Weiss, A. C. G.; McConville, C. F.; Padua, A.; Winkler, D. A.; Costa Gomes, M.; Greaves, T. L.; Le, T. C.; Besford, Q. A.; Christofferson, A. J. Systematic Comparison of the Structural and Dynamic Properties of Commonly Used Water Models for Molecular Dynamics Simulations. *J. Chem. Inf. Model.* **2021**, *61*, 4521–4536. https://doi.org/10.1021/acs.jcim.1c00794.
